# Structural, Functional, and Metabolic Brain Differences as a Function of Gender Identity or Sexual Orientation: A Systematic Review of the Human Neuroimaging Literature

**DOI:** 10.1007/s10508-021-02005-9

**Published:** 2021-05-06

**Authors:** Alberto Frigerio, Lucia Ballerini, Maria Valdés Hernández

**Affiliations:** Division of Health Sciences, University of Edinburgh, Edinburgh, EH16 4SB UK

**Keywords:** Gender identity, Neuroimaging, Sexual orientation, Transgender, Transsexual

## Abstract

This review systematically explored structural, functional, and metabolic features of the cisgender brain compared with the transgender brain before hormonal treatment and the heterosexual brain compared to the homosexual brain from the analysis of the neuroimaging literature up to 2018, and identified and discussed subsequent studies published up to March 2021. Our main aim was to help identifying neuroradiological brain features that have been related to human sexuality to contribute to the understanding of the biological elements involved in gender identity and sexual orientation. We analyzed 39 studies on gender identity and 24 on sexual orientation. Our results suggest that some neuroanatomical, neurophysiological, and neurometabolic features in transgender individuals resemble those of their experienced gender despite the majority resembling those from their natal sex. In homosexual individuals the majority resemble those of their same-sex heterosexual population rather than their opposite-sex heterosexual population. However, it is always difficult to interpret findings with noninvasive neuroimaging. Given the gross nature of these measures, it is possible that more differences too subtle to measure with available tools yet contributing to gender identity and sexual orientation could be found. Conflicting results contributed to the difficulty of identifying specific brain features which consistently differ between cisgender and transgender or between heterosexual and homosexual groups. The small number of studies, the small-to-moderate sample size of each study, and the heterogeneity of the investigations made it impossible to meta-analyze all the data extracted. Further studies are necessary to increase the understanding of the neurological substrates of human sexuality.

**Supplementary Information:**

The online version contains supplementary material available at 10.1007/s10508-021-02005-9.

## Introduction

### Sex, Gender Identity, and Sexual Orientation

Human sexuality is a complex and multilevel structure made up of different components, and it is usually described by different perspectives and using different terminologies. Despite the terms sex and gender being used interchangeably, we refer “sex” to the biological condition (chromosomal, gonadal, and phenotypic), “gender” to the inner psychological perception of one’s own identity (gender identity) and to the outer cultural perception in behavior and habits attributed to and assumed by masculinity and femininity (gender role), and “sexual orientation” to sexual attraction (sexual preference) (Shah et al., 2012).

The search for the origin of gender identity and sexual orientation is part of the debate on the impact of nature and culture on human life (Lippa, 2002). This topic is highly controversial, due to its cultural, social, and political implications, and it is widely debated within the scientific community. Despite the efforts of scientists in conducting objective research, research on social problems are influenced by the cultural environment, and often reflect the dominant theories of their time (Jordan-Young, 2010). The *vexata quaestio* is: To what extent are gender and sexual orientation biologically determined and/or socially constructed by personal experiences and cultural expectations?

On the one hand, the “born that way theory” holds that gender identity and sexual orientation are innate and fixed properties (Savic et al., 2010; Swaab, 2007, 2008). In this sense, brains of transgender and homosexual individuals would differ from brains of cisgenders and heterosexuals (Burke et al., 2017) in areas related to body perception (Savic & Arver, 2011) and sexual arousal (Sylva, 2013). In this framework, some authors refer to an early programming of gender identity and sexual inclinations due to alterations in sexual differentiation in the brain, decoupled from genital differentiation, as in transgenderism, or reduced, as in homosexuality. They propose that such altered sexual differentiation in the brain causes an alteration in the development of the brain areas modulating body perception (in transgenderism) or sexual arousal (in homosexuality) (Burke et al., 2017). On the other hand, the so-called gender theory holds that gender identity and sexual orientation are just cultural constructions, and it denies any kind of biological influence (Butler, 1990).

Actually, both gender identity and sexual orientation seem to develop under two main types of influence: biological (genes, hormones, and gene expression) and environmental (influences of parents, peers, partners, and social models) factors (Altinay & Anand, 2020; Balthazar, 2016; Jorge, 2010) as a result of the interaction between nature and culture (Hines, 2004). Evidence seems to suggest that biology contributes significantly to the development of both gender identity and sexual orientation (Roselli, 2018). Nevertheless, the idea that human sexuality is not biologically fixed is supported by longitudinal studies, which reported a certain fluidity in both gender identity (Drummond et al., 2008) and sexual orientation (Savin-Williams and Ream, 2007). Eventually, research on gender identity and sexual orientation is difficult because of the specificity of human sexuality, which makes difficult the use of animal models. Unlike animals, humans express their gender identity (Herbert, 2008), and their sexual behavior is, then, influenced by personal and social experiences and expectations (Maney, 2016).

### Studies on Gender Identity and Sexual Orientation

To verify whether and which biological factors are involved in the development of gender identity and sexual orientation, several studies have been conducted comparing cisgenders vs. transgenders, and heterosexuals vs. homosexuals. These studies allow the verification of whether or not there are specific features which could be related to the development of transgender identity and same-sex attraction as opposed to cisgender identity and heterosexual orientation. In this case, they would allow researchers to infer which elements are involved in the development of gender identity and sexual orientation as a whole. But let us first clarify the relevant terminology.

While the term cisgender refers to people whose sense of gender identity corresponds to their birth sex, the term transgender refers to individuals who identify themselves with the gender opposite to that assigned at birth. If transgenders ask for a hormonal and/or surgical affirmation, they are called transsexuals (APA, 2013). While the term heterosexual refers to people who are emotionally, romantically or sexually attracted to people of the opposite sex, the term homosexual refers to people who feel an emotional, romantic or sexual attraction toward subjects of the same sex (APA, 2008).

It is also important to distinguish between early-onset vs. late-onset transgenderism. While early-onset transgenderism appears before puberty (i.e., during infancy), late-onset transgenderism appears after puberty (i.e., adolescence or adult age). The early-onset form has been reported associated with a same-sex sexual orientation and referred to androphilia in birth-assigned males and gynephilia in birth-assigned females; while in the late-onset form, which is more common in birth-assigned males than in birth-assigned females, heterosexual orientation is not uncommon (Lawrence, 2010).

The neural bases of gender identity and sexual orientation have been studied through neural, hormonal, and genetic investigations. Post mortem studies reported brain differences between cisgender and transgender people (Garcia-Falgueras & Swaab, 2008; Kruijver et al., 2000; Zhou et al., 1995) and between heterosexual and homosexual subjects (Allen & Gorski, 1992; LeVay, 1991; Swaab & Hofman, 1990). Hormonal research suggests the involvement of prenatal hormones in the development of transgender identity (Cohen-Kettenis, 2005; Dessens et al., 2005) and homosexual orientation (McFadden, 2002; Zucker et al., 1996). Genetic investigations suggest a possible hereditary component for transgenderism (Green, 2000; Heylens et al., 2012; Segal, 2006; Veale et al., 2010) and homosexuality (Drabant et al., 2012; Wijchers & Festenstein, 2011). Overall, biological factors seem to play a role in shaping both gender identity and sexual orientation. Nevertheless, no evidence allows experts to conclude that they are determined by any specific factor, and many scientists think that both biological and social factors are involved in the development of gender identity (APA, 2014) and sexual orientation (APA, 2008).

A new frontier in research is represented by the neuroimaging techniques. A recent study shows pubertal testosterone being related to structural properties of the cerebral cortex (Liao et al., 2021). Imaging approaches focused on socio-emotional processing, executive functioning, and self-concept/image domains have been also recommended to study neurodevelopmental effects in transgenderism (Chen et al., 2020). With regard to gender identity, four studies have non-systematically discussed the results offered by neuroimaging. It has been pointed out that, before hormonal treatment, in transgenders the most important brain parameters, namely intracranial, gray matter, white matter, and cerebrospinal volumes, tend to be congruent with the gender assigned at birth—after hormone treatment they partly adjust to the characteristics of the desired gender -, although some structural, functional, and metabolic brain features may exhibit signs of masculinization or feminization (Guillamon et al., 2016; Kreukels & Guillamon, 2016; Mueller et al., 2017; Smith et al., 2015). With regard to sexual orientation, the neuroimaging literature is scarce. Investigations have reported structural (Abé et al., 2014; Manzouri & Savic, 2018; Ponseti et al., 2007; Savic & Lindström, 2008; Witelson et al., 2008), functional (Hu et al., 2008, 2011, 2013, 2014; Kagerer et al., 2011; Manzouri & Savic, 2018; Paul et al., 2008; Perry et al., 2013; Ponseti et al., 2009; Safron et al., 2017, 2018; Sylva, 2013; Zeki & Romaya, 2010; Zhang & Meaney, 2010), and metabolic (Berglund et al., 2006; Kinnunen et al., 2004; Savic & Lindström, 2008; Savic et al., 2005) differences between heterosexual and homosexual individuals, but an attempt to summarize and analyze these reports is, to the best of our knowledge, nonexistent.

Overall, neuroimaging investigations on both gender identity and sexual orientation have reported conflicting results, with considerable overlap between transgender or homosexual people and control population. The lack of systematically extracted data limits the progress in these areas of research. We conducted a systematic review and meta-analysis to investigate whether or not there are structural, functional, and metabolic neuroimaging features that differentiate cisgender from transgender and heterosexual from homosexual individuals, in an attempt to provide the scientific community data gathered from the whole body of scientific literature that has been produced up to date, extracted and uniformly processed.

### Aim

To document the scientific evidence from neuroimaging techniques on brain features that might be distinctive in cisgenders compared to transgenders (gender identity investigation), and in heterosexuals compared to homosexuals (sexual orientation investigation).

## Method

### Systematic Literature Search

A literature search was systematically conducted according to PRISMA (preferred reporting items for systematic reviews and meta-analyses) guidelines (Liberati, 2009). The search strategy, conducted in three different databases (Embase, Medline, PsycInfo), included articles published up to January 2018 comparing cisgenders vs. transgenders and articles published up to April 2018 comparing heterosexuals vs. homosexuals. Further, the initial search was updated with articles indexed in Medline from April 2018 up to March 2021.

Search terms used for the comparison between cisgenders and transgenders were brain AND (transgender OR transsexual OR gender dysphoria) AND (magnetic resonance imaging OR MRI OR diffusion tensor imaging OR DTI OR voxel-based morphometry OR VBM OR functional emission tomography OR fMRI OR positron emission tomography OR PET OR single photon emission computer tomography OR SPECT). Search terms used for the comparison between heterosexuals and homosexuals were brain AND (homosexual OR gay OR lesbian) AND (magnetic resonance imaging OR MRI OR diffusion tensor imaging OR DTI OR voxel-based morphometry OR VBM OR functional emission tomography OR fMRI OR positron emission tomography OR PET OR single photon emission computer tomography OR SPECT).

We analyzed only articles written in English and which published primary research output. Primary selection used title and abstract information. Authors were contacted if articles were not available online and/or if there was a question about the data presented in the article. After the initial selection, articles were checked for inclusion/exclusion criteria, and references were checked for possible further inclusions.

### Selection Criteria

#### Inclusion Criteria

The analysis of gender identity included articles which compared cisgender (non-transgender) population (male control = MC; female control = FC) with transgender people (male-to-female = MtF; female-to-male = FtM) before hormonal treatment, while the analysis of the sexual orientation included articles which compared heterosexual people (heterosexual man = HeM; heterosexual woman = HeW) with homosexual subjects (homosexual man = HoM; homosexual woman = HoW).

#### Exclusion Criteria

Articles that investigated people affected by neurological diseases or by diseases associated with neurological outcome (e.g., HIV) were not included. As hormonal treatment may affect brain features (Rametti et al., 2012), studies and/or data on transsexuality after hormonal treatment were excluded.

### Data Extraction

All quantitative outcomes including effect size and level of significance, regardless of whether or not they represented significant differences or not, were extracted from all papers included in the primary search conducted up to 2018. In addition, we independently extracted sample size, subject characteristics, mean age of subject population, type of neuroimaging technique (including field strength in the case of MRI), and regions of interest (ROI). Stereotaxic coordinates of activated/relevant brain areas were extracted from studies that used fMRI (resting state or not) and voxel-based morphometry (VBM). The findings from the papers identified in the period from April 2018 to March 2021 were summarized at the end of each correspondent subsection but not considered for the meta-analyses.

### Data Analysis

Microsoft Excel 2016 was used to represent the distribution of the demographic data and imaging modalities from all studies. The data extracted from each ROI were tabulated and visualized to draw conclusions. GingerAle 2.3.6 software was used to meta-analyze the stereotaxic coordinates that showed relevance to our research question for those studies that provided this information (i.e., those that used fMRI and VBM).

Due to the low number of studies conducted with metabolic neuroimaging techniques (i.e., PET and SPECT), it was not possible to carry out a meta-analysis of the brain regions that could metabolically differ between the groups of individuals involved in both of the analyses. The number of studies that used brain structural MRI to explore brain characteristics in relation to sexual orientation was also reduced, not allowing to meta-analyze these data either. Instead, we summarized this information.

To calculate the risk of bias within and across studies, we used the Quadas tool (Whiting et al., 2003). Quantitative results were converted to OR and CI using Practical Meta-Analysis Effect Size Calculator by Wilson (http://www.campbellcollaboration.org/escalc/html/EffectSizeCalculator-Home.php). After extracting all data available, it was not possible to do a meta-analysis per brain area due to the low number of studies with numerical data (see http://dx.doi.org/10.7488/ds/2412).

## Results

### Primary Literature Search

**Fig. 1:**
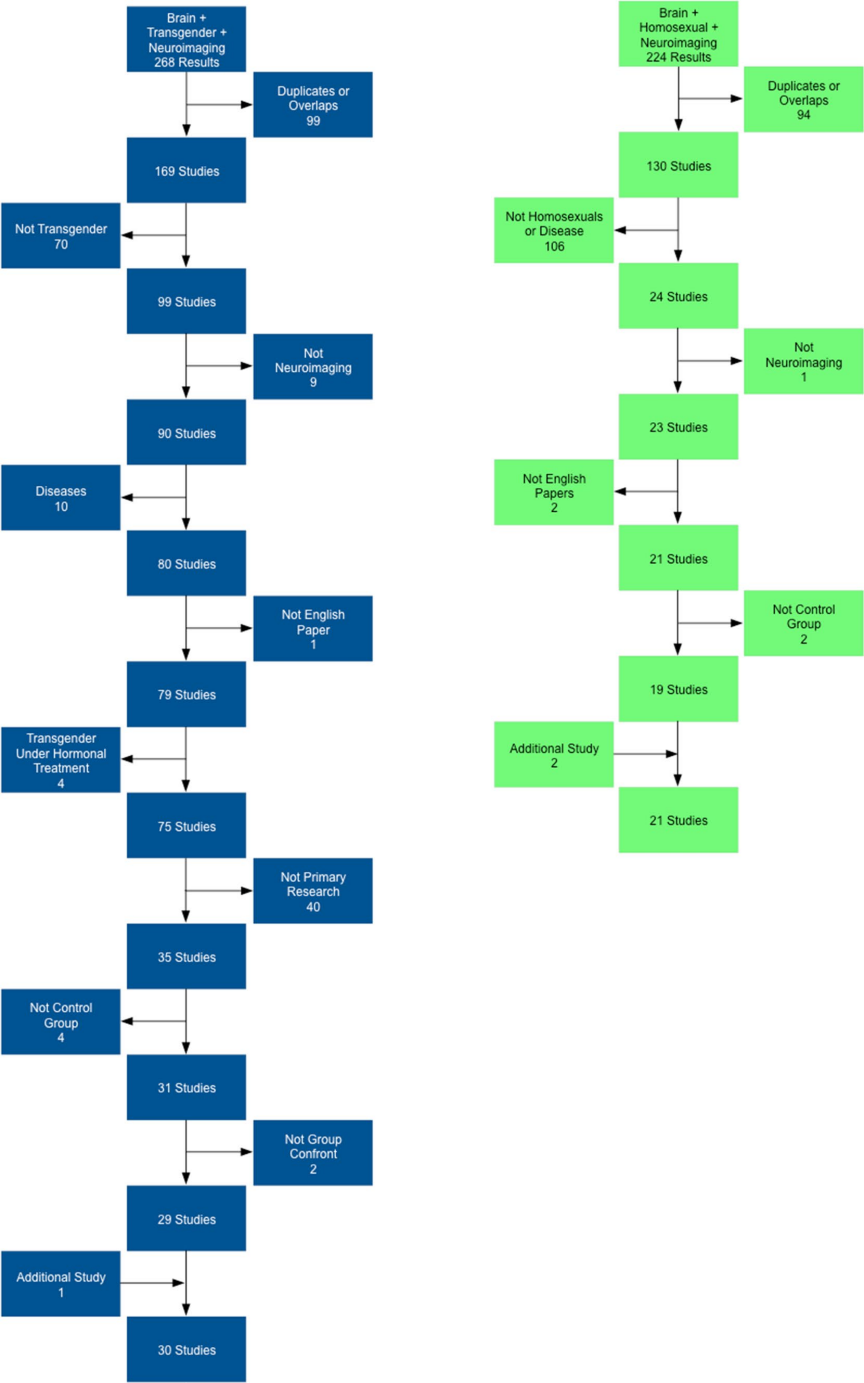
Flowcharts summarizing the study selection process for the analyses on gender identity (left) and sexual orientation (right)

**Fig. 2:**
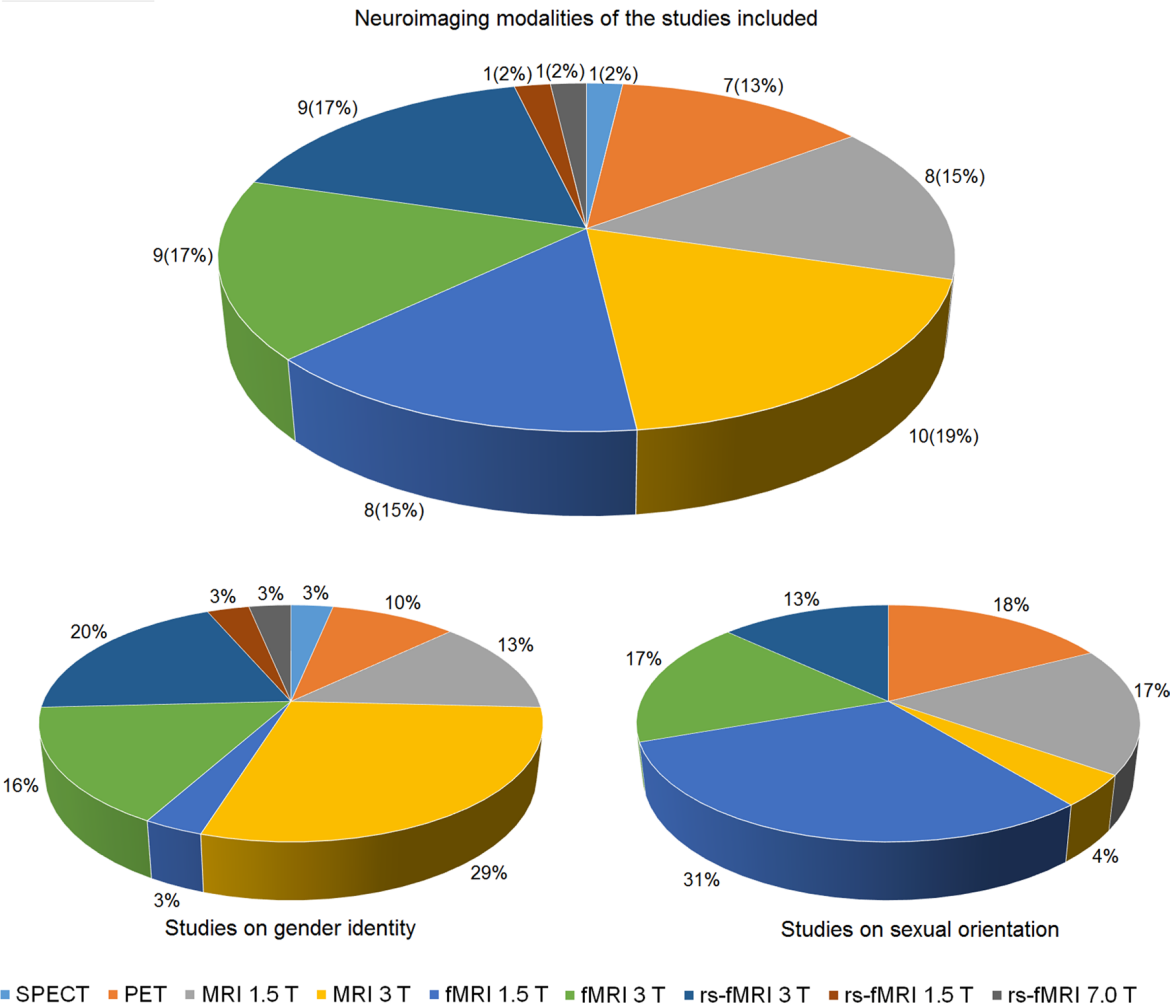
3D pie charts summarizing the number and percentage of studies included in the analyses, conducted with different neuroimaging techniques: overall information (top), analysis on gender identity (bottom left), and analysis on sexual orientation (bottom right)

**Fig. 3:**
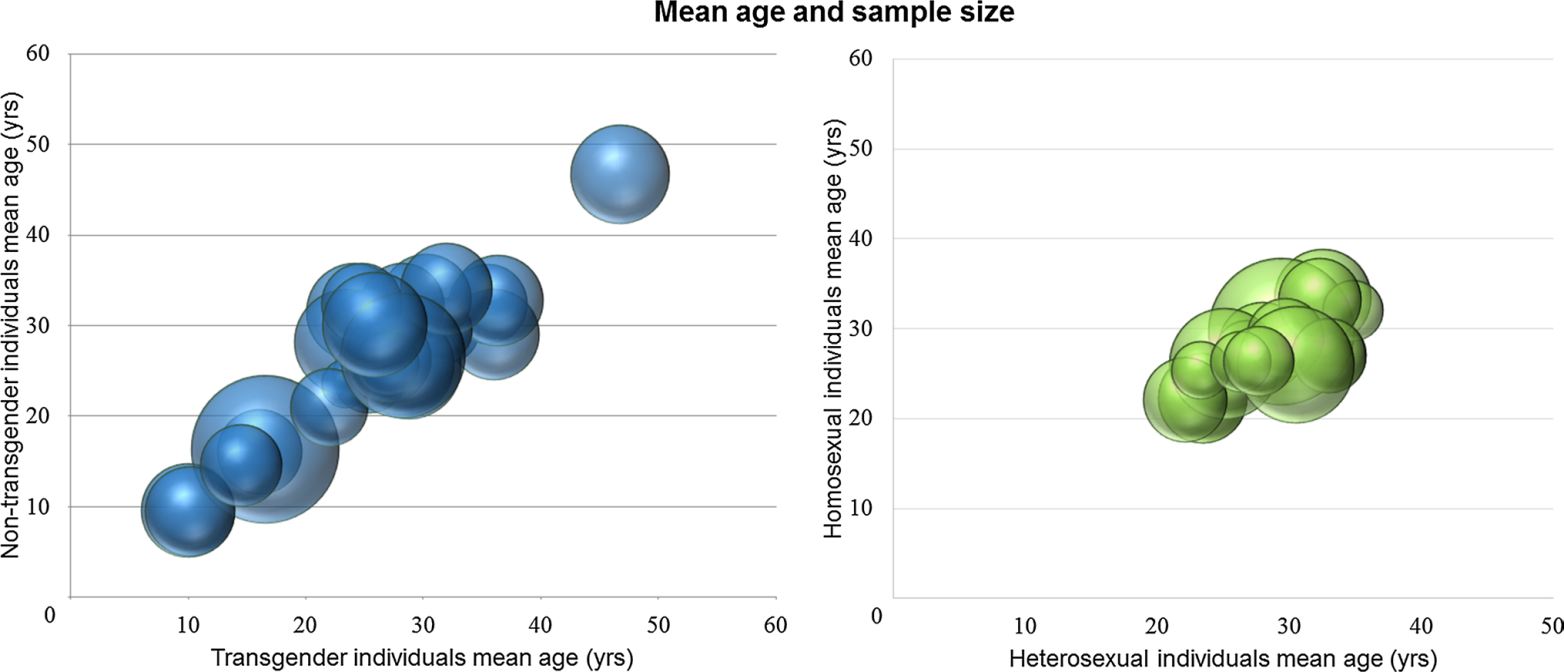
3D bubble charts of the mean age and sample size of: **a** cisgenders and transgenders involved in the selected studies (left) (Note: the graph does not include the study by Yokota et al., 2005 because of the lack of data), and **b** heterosexuals and homosexuals involved in the selected studies (right) (Note: the graph does not include the study by Hu et al., 2011 because of the lack of data. Four studies (Kagerer et al., 2011; Perry et al., 2013; Sylva, 2013; Zeki & Romaya, 2010) reported just the mean age of all the sample size, and we assumed that it was the same in heterosexual and homosexual subsamples)

The systematic search up to 2018 generated 492 publications from the three different databases: 268 for the analyses of gender identity and 224 for the analyses on sexual orientation. A total of 51 studies were included: 30 for the analyses of gender identity and 21 for the analyses on sexual orientation (Fig. 1 and Appendices 1 and 2). All studies were conducted using different neuroimaging techniques: structural (MRI), functional (fMRI and rs-fMRI), and metabolic (PET and SPECT) (Fig. 2). The majority of the studies included used functional MRI (28/51 studies: 61% of the studies on sexual orientation and 45% of the studies on gender identity). Studies that used PET and SPECT modalities were few in both analyses (13% of the studies on gender identity and 18% of the studies on sexual orientation). Figure 3 shows the mean age and sample size of the groups of individuals involved in the analyses. The analysis on gender identity involved individuals across a wider age range (mean ages 9.5–46.7 years old, i.e., including two studies on gender identity in children in pre-puberty age (9–10 years old) and three in adolescents (14–16 years old)) than the analysis on sexual orientation (mean ages 22.1–33.4 years old), but none of them covered early infancy and neither later adulthood. Due to the small number of studies included and the heterogeneity in the information available (i.e., from the included studies), we did not classify the papers with respect to the homogeneity of the groups (i.e., in terms of social background, education, comorbidities, genetic and risk factors of the individuals involved).

### Gender Identity Analyses: Study Selection

From 268 publications, 99 papers were duplicate or overlapped, 29 papers matched the inclusion criteria, and 140 were excluded. An additional study was included from the references (see Table 1 for study information).

**Table 1:** Studies included in the analysis of gender identity

Study: First author’s surname (if one or more than two authors)	Year	Sample	Mean age	Technique
Berglund	2008	12 MtF–12 MC–12 FC	32–26–33	PET
Burke	2014	17 FtM–19 MtF–19 FC–20 MC	9.6–10.4–9.7–9.5	fMRI
Burke	2016	21 FtM–21 FC–20 MC	16.1–16.3–15.9	fMRI
Clemens	2017	15 MtF–21 MC–20 FC	35.5–32.32–32.5	rs-fMRI
Feusner	2017	27 FtM–27 FC–27 MC	24.2–32.1–31	rs-fMRI
Gizewski	2009	12 MtF–12 MC–12 FC	36–29–29	fMRI
Hahn	2015	23 FtM–21 MtF–25 FC–25 MC	26.9–30.9–25.3–25.6	MRI
Hoekzema	2015	54 FtM–37 MtF–52 FC–44 MC	16.92–16.05–16.29–16.42	MRI
Junger	2014	16 MtF–21 MC–20 FC	36.38–32.35–33.16	fMRI
Kranz	2014a	14 MtF–13 MC–9 FC	31.4–29.8–29	PET
Kranz	2014b	23 FtM–21 MtF–23 FC–22 MC	25.91–30.86–25.96–25.45	MRI
Kranz	2015	14 FtM–19 MtF–11 FC–24 MC	28.21–31.79–30.43–34.14	PET
Kranz	2018	25 FtM–12 FC–13 MC	27.24–24.42–28.77	MRI
Ku	2013	12 FtM–11 MtF–12 FC–11 MC	All Trans 25.4–All Cis 24.4	rs-fMRI
Lin	2014	12 FtM–11 MtF–12 FC–11 MC	All Trans 25.4–All Cis 24.4	rs-fMRI
Luders	2009	24 MtF–30 MC–30 FC	46.73–46.57–46.77	MRI
Manzouri	2017	28 FtM–34 FC–34 MC	23.5–27.6–28.8	MRI and rs-fMRI
Nawata	2010	11 FtM–9 FC	23.4–23.6	SPECT
Nota	2017	13 FtM–18 MtF–18 FC–21 MC	9.7–10.5–9.6–9.4	rs-fMRI
Pol	2006	6 FtM–8 MtF–6 FC–9 MC	28–25–23–25	MRI
Rametti	2011a	18 FtM–19 FC–24 MC	28.24–31.22–33	MRI
Rametti	2011b	18 MtF–19 MC–19 FC	24.74–31.94–33	MRI
Santarnecchi	2012	1 FtM–25 FC–25 MC	22–21–21	rs-fMRI
Savic and Arver	2011	24 MtF–24 MC–24 FC	32–33–35	MRI
Schöning	2010	11 MtF–11 MC	37.55–33.09	fMRI
Simon	2013	7 FtM–10 MtF–7 FC–11 MC	24.8–28.5–23.9–27.1	MRI
Soleman	2013	11 FtM–6 MtF–26 FC–24 MC	14.64–14.25–14.44–14.68	fMRI
Spies	2016	33 FtM–24 MtF–44 FC–33 MC	26.79–30.25–26.16–27.48	rs-fMRI
Yokota	2005	28 FtM–22 MtF–211 FC–211 MC	Not reported	MRI
Zubiaurre-Elorza	2013	24 FtM–18 MtF–23 FC–29 MC	26.21–25.5–31.09–29.28	MRI

FtM, female-to male; MtF, male-to-female; FC, female control; MC, male control

### Sexual Orientation Analyses. Study Selection

From 224 publications, 94 papers were duplicate or overlapped, 19 papers matched the inclusion criteria, and 111 were excluded. Two additional studies were included (see Table 2 for study information).

**Table 2:** Studies included in the analysis of sexual orientation

Study reference	Year	Sample	Mean Age	Technique
Abé et al	2014	19 HoM–21 HeM–21 HeW	33.5–31.9–33.2	MRI
Berglund et al	2006	12 HoW–12 HeW–12 HeM	33–26–28	PET
Hu et al	2013	26 HoM–26 HeM	22.27–23.46	rs-fMRI
Hu et al	2014	26 HoM–26 HeM	22.27–23.46	rs-fMRI
Hu et al	2011	14 HoM–14 HeM	Not reported	fMRI
Hu et al	2008	10 HoM–10 HeM	26.5–27.9	fMRI
Kagerer et al	2011	11 HoM–10 HeM	All sample 28	fMRI
Kinnunen et al	2004	8 HoM–7 HeM	29–28	PET
Manzouri-Savic	2018	30 HoM–30 HoW–40 HeM–40 HeW	31.4–27.9–29.5–29.3	MRI and rs-fMRI
Paul et al	2008	12 HoM–12 HeM	32–34.8	fMRI
Perry et al	2013	12 HoM–12 HoW–13 HeM–15 HeW	All sample 28.46	fMRI
Ponseti et al	2009	14 HoM–12 HeM	27.4–26.8	fMRI
ponseti et al	2007	16 HoM–15 HoW–24 HeM–25 HeW	27.3–24.9–25.3–24.9	MRI [VBM]
Safron et al	2017	22 HoM–23 HeM 22 HoM–19 HeM	33.2–32.3 Not reported	fMRI [picture] fMRI [video]
Safron et al	2018	20 HoW–20 HeW 20 HoW–18 HeW	29–29.7 Not reported	fMRI [picture] fMRI [video]
Savic et al	2005	12 HoM–12 HeM–12 HeW	33–28–26	PET
Savic-Lindström	2008	20 HoM–20 HoW–25 HeM–25 HeW 12 HoM–12 HoW–13 HeM–13 HeW	32–20–30–31 Not reported	MRI PET
Sylva et al	2013	12 HoM–11 HoW–12 HeM–11 HeW	All sample 22.1	fMRI
Witelson et al	2008	12 HoM–10 HeM	25.4–23.3	MRI
Zeki-Romaya	2010	6 HoM–6 HoW–6 HeM–6 HeW	All sample 26.3	fMRI
Zhang et al	2011	16 HoM–16 HeM	26.4–27.7	fMRI

HoM, homosexual men; HoW, homosexual women; HeM, heterosexual men; HeW, heterosexual women

### Update from April 2018 until March 2021

From 86 papers identified from the literature in this short period, 9/74 publications in gender identity and 3/12 publications on sexual orientation matched our inclusion/exclusion criteria.

### Regions of Interest Analyses

#### Gender Identity

Structural MRI was conducted in 13 of the studies published up to 2018. However, only one of them conducted the analysis in specific stereotaxic coordinates (Simon et al., 2013). The 12 studies that conducted ROI analysis involved 229 FtM, 169 MtF, 478 FC, and 484 MC. Table 3 shows the ROI and the parameters investigated by each of these studies. Two studies involving 79 out of 229 FtM, 37 out of 169 MtF, 64 out of 478 FtM, and 57 out of 484 MtF did not find differences in the mean diffusivity of the hypothalamus (Kranz et al., 2018) nor in the volumes of cerebellum, hypothalamus, and medial frontal cortex (Hoekzema et al., 2015). Differences between cisgenders and transgenders were reported in 10/12 studies in white matter microstructure (four studies), volumetric analysis (four studies), cortical thickness (two studies), and corpus callosum shape (one study).

**Table 3:** Regions of Interest analysis on gender identity

Reference	ROI	Parameters	Trans_vs_Natal Sex	Trans_vs_Opposite Sex	Trans_vs_Cis	M_vs_F
Hahn et al. (2015)	Subcortical L	HCR	Nr	Nr	Nd	Nd
	Subcortical R	HCR	Nr	Nr	v	v
	Subcortical R–frontal R	LCW	Nr	Nr	Nd	Nd
	Subcortical L–parietal L	LCW	Nr	Nr	Nd	Nd
Hoekzema et al. (2015)	Cerebellum L	Volume	Nd	Nr	Nr	v
	Cerebellum R	Volume	Nd	Nr	Nr	v
	Hypothalamus	Volume	x	Nr	Nr	v
	Medial frontal cortex	Volume	Nd	Nr	Nr	v
Kranz et al. (2018)	Hypothalamus	MD	x	x	x	v
Kranz et al. (2014b)	GM	Volume	x (MtF)–x (FtM)	v (MtF)–v (FtM)	x	v
	WM	Volume	x (MtF)–x (FtM)	v (MtF)–v (FtM)	x	v
	CSF	Volume	x (MtF)–x (FtM)	v (MtF)–v (FtM)	x	v
	TIV	Volume	x (MtF)–x (FtM)	v (MtF)–v (FtM)	x	v
	CST R	MD	v (MtF)–v (FtM)	v (MtF)–v (FtM)	v	v
	CST L	MD	v (MtF)–v (FtM)	v (MtF)–v (FtM)	v	v
	Forceps major	MD	x (MtF)–x (FtM)	x (MtF)–x (FtM)	x	x
	Forceps minor	MD	v (MtF)–v (FtM)	v (MtF)–v (FtM)	v	v
	CST R	FA	x (MtF)–x (FtM)	x (MtF)–x (FtM)	x	x
	CST L	FA	x (MtF)–x (FtM)	x (MtF)–x (FtM)	x	x
	Forceps major	FA	x (MtF)–x (FtM)	x (MtF)–x (FtM)	x	x
	Forceps minor	FA	v (MtF)–v (FtM)	v (MtF)–v (FtM)	x	x
Luders et al. (2009)	Frontal lobe	Volume	x	v	Nd	v
	Occipital lobe	Volume	x	v	Nd	v
	Parietal lobe	Volume	x	v	Nd	v
	SFG	Volume	x	v	Nd	v
	Midline	Volume	x	v	Nd	v
	Frontal pole	Volume	x	v	Nd	v
	Caudate nucleus	Volume	x	v	Nd	v
	Putamen	Volume	v	x	Nd	v
	Subcallosum gyrus	Volume	x	v	Nd	v
	Mammillary body	Volume	x	v	Nd	v
	Amygdala	Volume	x	v	Nd	v
	Thalamus	Volume	x	v	Nd	v
	Hypothalamus	Volume	x	v	Nd	v
	Basal surface	Volume	x	v	Nd	v
Manzouri et al. (2017)	Cortex	CTh	v	v	v	v
	Surface	SA	x	v	Nd	v
	GM	Volume	x	v	Nd	v
	Hippocampus R	Volume	x	v	Nd	v
	Hippocampus L	Volume	x	v	Nd	v
	Thalamus R	Volume	x	v	Nd	v
	Thalamus L	Volume	x	x	x	x
	Caudate R	Volume	x	v	Nd	v
	Caudate L	Volume	x	x	x	x
	Putamen R	Volume	x	v	Nd	v
	Putamen L	Volume	v	v	v	v
	Amygdala R	Volume	x	x	x	x
	Amygdala L	Volume	x	v	Nd	v
	Cerebellum R	Volume	x	x	x	x
	Cerebellum L	Volume	x	x	x	x
	Pallidum R	Volume	x	x	x	x
	Pallidum L	Volume	x	x	x	x
	Total intracranial	TIV	x	v	Nd	v
Pol et al. (2006)	Intracranial	Volume	x	v	x	v
	Total brain	Volume	x	v	x	v
	Hypothalamus	Volume	x	x	x	x
	3rd ventricle	Volume	x	x	x	x
	Lateral ventricle	Volume	x	x	x	x
	GM	Volume	x	x	x	x
	WM	Volume	x	x	x	x
Rametti et al. (2011a)	SLF R	FA	v	x	Nd	v
	Forceps minor	FA	v	x	Nd	v
	CST	FA	v	v	v	v
Rametti et al. (2011b)	GM	Volume	x	v	Nd	v
	WM	Volume	x	v	Nd	v
	CSF	Volume	x	v	Nd	v
	Total intracranial	TIV	x	v	Nd	v
	SLF R	FA	v	v	v	v
	Forceps minor	FA	v	v	v	v
	CST	FA	v	v	v	v
	Anterior Cingulum R	FA	v	v	v	v
Savic and Arver (2011)	Hippocampus	Volume	x	Nd	Nd	v
	Thalamus	Volume	v	v	v	x
	Caudate	Volume	x	x	x	x
	Putamen	Volume	v	v	v	x
	Total tissue	Volume	x	Nd	Nd	v
	Total brain	Volume	x	x	x	x
	GM	Volume	x	x	x	x
	WM	Volume	x	x	x	x
Yokota et al. (2005)	Corpus callosum	Shape	v	v	v	v
Zubiaurre-Elorza et al. (2013)	Cortex	CTh	v (MtF)–x (FtM)	x (MtF)–v (FtM)	Nd	v
	Putamen R	Volume	x (MtF)–v (FtM)	x (MtF)–x (FtM)	Nd	v
	Putamen L	Volume	x (MtF)–x (FtM)	x (MtF)–x (FtM)	x	Nr
	Thalamus	Volume	x (MtF)–x (FtM)	x (MtF)–x (FtM)	x	Nr
	Caudate	Volume	x (MtF)–x (FtM)	x (MtF)–x (FtM)	x	Nr
	Pallidum	Volume	x (MtF)–x (FtM)	x (MtF)–x (FtM)	x	Nr
	Hippocampus	Volume	x (MtF)–x (FtM)	x (MtF)–x (FtM)	x	Nr
	Amygdala	Volume	x (MtF)–x (FtM)	x (MtF)–x (FtM)	x	Nr

Cis, natal and opposite sex; GM, gray matter; WM, white matter; CSF, cerebrospinal fluid; CST, corticospinal tract; SFG, superior frontal gyrus; SLF, superior longitudinal fasciculus; R, right; L, left; HCR, hemispheric connectivity ratio; LCW, lobar connectivity weight; MD, mean diffusivity; FA, fractional anisotropy; CTh, cortical thickness; SA, surface area; GMV, gray matter volume; TIV, total intracranial volume; v, differences; x, non differences; Nd, not definable; Nr, not reported

*White matter microstructure* of cisgender and transgender groups was analyzed by four studies. Only one study (23 FtM, 21 MtF, 25 FC, and 25 MC) investigated the structural connectome, which is the complete map of the neural connections in a brain, and found differences between cisgender and transgender population in the right subcortical hemispheric connectivity ratio (Hahn et al., 2015). Three studies analyzed fractional anisotropy (FA) and mean diffusivity (MD), of which only one involving 18 FtM, 19 FC, and 24 MC found that in FtM FA was masculinized in some brain areas (right superior longitudinal fasciculus and in the forceps minor) and fell halfway between male and female patterns in other brain areas (corticospinal tract) (Rametti et al., 2011a). The other two studies either did not find significant differences in FA between different groups (Kranz et al., 2014b) (involved 23 FtM, 21 MtF, 23 FC, and 22 MC) or found that MtFs FA fell halfway between MC and FC pattern in some brain regions (right superior longitudinal fasciculus, forceps minor, corticospinal tract, right anterior cingulum) (Rametti et al., 2011b) (involved 18 MtF, 19 MC, 19 FC). MD in transgender groups (either MtF or FtM) was found to fall halfway between FC and MC people in corticospinal tract right and left and in forceps minor by one study that involved 23 FtM, 21 MtF, 23 FC, 22 MC (Kranz et al., 2014b).

*Subcortical gray matter volume* was investigated by four studies. All of them found that the volume of the putamen was consistently different between cisgender and transgender groups (Luders et al., 2009; Manzouri et al., 2017; Savic & Arver, 2011; Zubiaurre-Elorza, et al., 2013). Manzouri et al. (2017) found that the FtMs left putamen was larger than both female and male cisgenders (sample: 28 FtM, 34 FC, 34 MC), and Savic and Arver (2011) found that among all subcortical structures, MtF’s putamen and thalamus were smaller than those in both female and male cisgender groups (sample: 24 MtF, 24 MC, 24 FC). These two studies also found that total gray matter volume did not differ between the transgender and cisgender groups. Zubiaurre-Elorza et al. (2013) also investigated subcortical gray matter in 24 FtM, 18 MtF, 23 FC and 29 MC and reported that FtM had atypically larger right putamen, compared to the typical size of this brain structure in FC in average. Luders et al. (2009) investigated gray matter volumes in 22 different regions, 12 in the right hemisphere, and 10 in the left hemisphere (i.e., frontal, occipital and parietal lobes, superior frontal gyrus, midline, frontal pole, basal ganglia—caudate nucleus and putamen -, limbic system—subcallosum gyrus, mammillary body, amygdala, thalamus, hypothalamus, basal surface), in 24 MtF, 30 MC, 30 FC, and found putaminal volume in MtF to be atypically smaller (i.e., compared to the typical average putaminal volume in MC).

*Cortical thickness* was investigated in two studies, which reported differences between cisgender and transgender groups in only a few non-overlapping regions. One (28 FtM, 34 FC, 34 MC) found differences between FtM and both FC and MC in the supramarginal, parietal, rostral middle frontal, inferior temporal gyrus, superior frontal gyrus, and lingual-precalcarine cortex cuneus (Manzouri et al., 2017), and the other (24 FtM, 18 MtF, 23 FC, 29 MC) reported that MtFs had orbitofrontal, medial occipital and insular regions that resemble those typically seen in the female control group (Zubiaurre-Elorza et al., 2013). Manzouri and Savic, in another study published in 2019 involving 27 FtM, 40 MtF, 40 heterosexual MC, 40 heterosexual FC, 30 homosexual MC and 30 homosexual FC found that cortical thickness did not differ between heterosexual controls and transgenders of the same birth-assigned sex. Transgender groups presented thicker clusters at the temporal and parietal cortex compared to heterosexual controls of their experienced gender, but these results were no longer observed when compared against homosexual controls. (Manzouri & Savic, 2019).

*Corpus callosum shape* was investigated by only one study (28 FtM, 22 MtF, 211 FC, 211 MC). It found that in transgender people it was closer to their experienced gender than to their assigned sex at birth (Yokota et al., 2005).

Four additional studies analyzing structural brain differences between cisgenders and transgenders were identified in the period between April 2018 and March 2021. A study published in 2020 involving 26 males and females aged 19–38 concludes that the nucleus accumbens, left thalamus, right hippocampus, and right caudate nucleus were smaller in transgenders (sample size 11) than in cisgenders (sample size 15). However, did not specify the biological sex of the transgender and cisgender samples (Starcevic et al., 2020). In a large study, also published in the same year involving 121 individuals (mean ages: 27.17 (23 MtF), 30.17 (29 FtM), 27.09 (34 MC), 26.29 (35 FC) years old) authors manifest impossibility to conclude whether the brain structure of the transgender groups resemble or not the morphology of their respective gender identity (Baldinger-Melich et al., 2020). This result is not surprising given that all sample groups involved non-balanced subsamples of individuals with different sexual orientation (i.e., heterosexuals, bisexuals and homosexuals). Two additional studies, one also published in 2020 involving 80 transgender and 60 cisgender non-western individuals (Khorashad et al., 2020), and another published in 2021 involving 16 FtM, 17 FC and 14 MC (Skorska et al., 2021) concluded that cortical morphometry (mainly surface area) was related to sex assigned at birth, but not to the experienced gender.

#### Sexual Orientation

Five MRI studies were analyzed (1/5 did not report the ROI analysis, but it provided stereotaxic coordinates using voxel-based morphometry). The four studies that conducted the ROI analysis involved 81 HoM, 50 HoW, 96 HeM, and 86 HeW.

Due to the low number of studies conducted with MRI, it was not possible to do a meta-analysis on structural features in homosexual subjects compared to heterosexual subjects. However, findings contained in these studies offer data worth describing. Table 4 shows the ROI and the parameters investigated by each of these studies.

**Table 4:** Regions of Interest analysis on sexual orientation

Reference	ROI	Prameters	Homo_vs_Hetero Natal Sex	Homo_vs_Hetero Opposite Sex	M_vs_F
Abé et al. (2014)	Middle temporal cortex L	CTh	x	v	v
	Superior temporal cortex L	CTh	x	x	v
	Inferior temporal cortex R	CTh	v	v	v
	Lateral orbitofrontal cortex R	CTh	v	x	v
	Pars Triangularis R	CTh	v	v	v
	Lingual cortex R	CTh	v	x	v
	Cuneus cortex R	CTh	v	x	v
	Pericalcarine cortex R	CTh	v	x	v
	Amygdala	Volume	x	v	v
	Cerebellum	Volume	x	v	v
	Hippocampus	Volume	x	v	v
	Putamen	Volume	x	v	v
	Thalamus	Volume	v	v	v
Manzouri and Savic (2018)	Parietal lobe cortex	CTh	v (HoM)–x (HoW)	x (HoM)–v (HoW)	v
	Superior temporal gyrus cortex	CTh	x (HoM)–x (HoW)	v (HoM)–x (HoW)	v
	Amygdala	Volume	x (HoM)–x (HoW)	v (HoM)–v (HoW)	v
	Caudate	Volume	x (HoM)–x (HoW)	x (HoM)–x (HoW)	x
	Hippocampus	Volume	x (HoM)–x (HoW)	v (HoM)–v (HoW)	v
	Putamen	Volume	x (HoM)–x (HoW)	x (HoM)–x (HoW)	x
	Total intracranial	TIV	x (HoM)–x (HoW)	x (HoM)–x (HoW)	x
	WM	FA	x (HoM)–x (HoW)	v (HoM)–v (HoW)	v
Savic and Lindström (2008)	Cerebral hemisphere R	Volume	v (HoM)–v (HoW)	x (HoM)–x (HoW)	v
	Cerebral hemisphere L	Volume	x (HoM)–x (HoW)	x (HoM)–x (HoW)	x
	Cerebellar hemisphere R	Volume	x (HoM)–x (HoW)	x (HoM)–x (HoW)	x
	Cerebellar hemisphere L	Volume	x (HoM)–x (HoW)	x (HoM)–x (HoW)	x
Witelson et al. (2008)	Anterior half CC	Volume	x	Nr	Nr
	Posterior mid-body CC	Volume	x	Nr	Nr
	Isthmus CC	Volume	v	Nr	Nr
	Mid-sagittal area CC	Volume	x	Nr	Nr
	Splenium CC	Volume	x	Nr	Nr
	Total CC	Volume	x	Nr	Nr

WM, white matter; CC, corpus callosum; CTh, cortical thickness; TIV, total intracranial volume; FA, fractional anisotropy; v, differences; x, non differences; Nr, Not reported

*Cortical thickness* (CTh) was investigated by two studies (Abé et al., 2014; Manzouri & Savic, 2018). While Abé et al. (2014) found that HoM have a thinner CTh than HeM in the visual area, Manzouri and Savic (2018) found that HoM have a thicker CTh than HeM in the parietal lobe. No significant differences were found between HoW and HeW.

*Subcortical volumes* were investigated by three studies (Abé et al., 2014; Manzouri & Savic, 2018; Witelson et al., 2008). Abé et al. (2014) found a smaller thalamus volume in HoM than HeM, while Witelson et al. (2008) found that HoM had a larger corpus callosum in the isthmus region. No other significant effects of sexual orientation were found.

A study measured *cerebral and cerebellar hemispheres* (Savic & Lindström, 2008). With regard to the cerebral hemisphere, they were symmetrical in HoM and in HeW, while they were asymmetrical in HoW and in HeM. With regards to the cerebellar hemisphere, no group had asymmetry. Another study investigated *white matter tracts* of the whole brain (Manzouri & Savic, 2018), and did not find significant differences between heterosexual and homosexual groups.

A diffusion tensor imaging study published in 2020 involving 53 homosexual and 47 heterosexual men found lower connectivity between left postcentral gyrus and left supramarginal gyrus in the homosexual group compared to the heterosexual group (Wang, Hu, et al., 2020).

### Stereotaxic Coordinates Analysis

#### Gender Identity

**Fig. 4:**
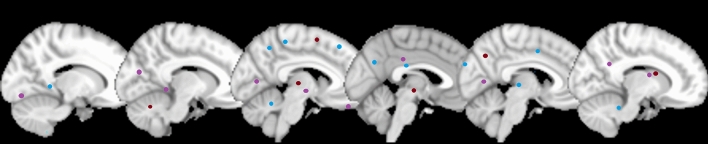
Six representative sagittal slices showing the foci resultant from the meta-analyses of stereotaxic coordinates where significant differences were found between groups with opposite gender identity (result from GingerAle 2.3.6; figure generated with micron.exe) (Purple = Transgender_vs_Natal sex; red = Transgender_vs_Opposite sex; blue = Transgender_vs_Natal and Opposite Sex)

Six fMRI, eight rs-fMRI, and three VBM studies published up to 2018 were considered for meta-analyses. fMRI studies were conducted under visual stimulation (2), smelling stimulation (1), vocal stimulation (1), a mental rotation task (1), and a verbal fluency test (1). The 17 studies that conducted stereotaxic coordinates analysis involved 195 FtM, 208 MtF, 347 FC, and 346 MC. Figure 4 displays six representative slices showing the foci resultant from the meta-analysis carried out using GingerAle 2.3.6 software using data from 12/17 studies (Burke et al., 2014, 2016; Clemens et al., 2017; Feusner et al., 2017; Gizewski et al., 2009; Hoekzema et al., 2015; Junger et al., 2014; Ku et al, 2013; Manzouri et al., 2017; Santarnecchi et al., 2012; Savic and Arver, 2011; Schöning et al., 2010; Simon et al, 2013) (see Appendix 3 in Supplementary Materials for the labels of each foci and Table 5 for the number of foci related to different brain areas). The meta-analyses conducted (“Transgender_vs_Cisgender Natal Sex”, “Transgender_vs_Cisgender Opposite Sex”, and “Transgender_vs_Cisgender”) showed that transgender people’s brain activation differed more frequently in the Brodmann Areas (BA) 18 and 19, which include the occipital visual area along with BA 17, which is involved in visual processing.

**Table 5:** Stereotaxic coordinates analysis on gender identity (number of foci related to different brain areas)

Transgender_vs_Cisgender Natal Sex		Transgender_vs_Cisgender Opposite Sex		Transgender_vs_Cisgender	
BA 18	7	BA 19	4	BA 18	9
Thalamus	2	Crebellum	2	BA 10	6
BA 10	2	BA 9	2	BA 19	6
BA 22	2	Insula	2	Insula	4
BA 23	2	Anterior cingulate	1	BA 32	4
Insula	1	Frontal gyrus	1	Thalamus	3
Caudate	1	Posterior cingulate	1	BA 22	3
Gyrus precuneus	1	Putamen	1	Brainstem	2
Hypothalamus	1	Thalamus	1	Cerebellum	2
Midbrain	1	BA 10	1	Frontal gyrus	2
Parahippocampal gyrus	1	BA 22	1	Hypothalamus	2
Perisylvian	1	BA 24	1	Posterior cingulate cortex	2
Substantia	1	BA 39	1	BA 9	2
BA 6	1			BA 31	2
BA 9	1			BA 23	1
BA 11	4			Anterior cingulate cortex	1
BA 17	3			Caudate	1
BA 19	2			Fusiform	1
BA 31	5			Hippocampus	1
BA 32	1			Perisylvian	1
				Precentral gyrus	1
				Putamen	1
				Temporal gyrus	1
				BA 4	1
				BA 5	1
				BA 7	1
				BA 8	1
				BA 21	1
				BA 24	1
				BA 37	1
				BA 40	1

BA, Brodmann area; Transgender_vs_Cisgender Natal Sex, MtF_vs_MC + FtM_vs_FC; Transgender_vs_Cisgender Opposite Sex, MtF_vs_FC + FtM_vs_MC

Five studies (1/6 fMRI and 4/8 rs-fMRI) did not report the stereotaxic coordinates, and and it was impossible to determine them in all but one case. A study (11 MtF, 12 FtM, 11 MC, and 12 FC) conducted with rs-fMRI investigated the resting state functional connectivity network and identified differences between cisgenders and transgenders in brain regions that seem to be involved in the neural network of body representation, and it concluded that different body representation may have different connectivity representation. However, it remains unclear whether or not a certain connectivity pattern is specific to transgenderism (Lin et al., 2014). A study (18 MtF, 13 FtM, 21 MC, 18 FC) conducted with rs-fMRI investigated functional connectivity patterns and reported that in pre-puberal children functional connectivity was similar in all groups (Nota et al., 2017), although stressed the necessity of increasing the sample to draw meaningful conclusions. Another study (24 MtF, 33 FtM, 33 MC, 44 FC) that also investigated resting state functional connectivity (i.e., also a rs-fMRI study) found significant differences within a network around the supramarginal gyrus (i.e., a subregion within the parietal lobe) (Spies et al., 2016). A study (8 MtF, 14 FtM, 25 MC, 26 FC) conducted with fMRI did not report any differences between transgender subjects and control group population during a verbal fluency test (Soleman et al., 2013). These four studies seem to suggest that in transgenders, brain activations have an intermediate pattern between those typical for their natal sex and experienced gender.

Four studies published in 2020 were additionally identified. An fMRI (emotional task) study on adolescents (mean age: 16.1 (FtM), 15.9 (HeM), 16.4 (HeW)) did not find differences in amygdala lateralization before hormonal (i.e., testosterone) treatment in FtM compared to HeM and HeW (Beking et al., 2020). A study comparing performance of 20 cisgender vs. 20 transgender individuals in gender face perception tasks showed bilateral activation differences in the precuneus of FtM compared to FC. MtF, in addition, significantly differed from MC in the lateral occipital cortex, posterior cingulate and angular gyri (Fisher et al., 2020). A study that conducted rs-MRI in young adults aged 19–22 years old, found MtF, FtM and FC having less resting state activations in parietal regions than MC; and MtF having also weaker functional connectivity in some regions in frontal cortex than MC (Uribe et al., 2020). Similar to the study from Spies et al. (2016), also in rs-fMRI, this study suggests that in MtF some parietal and frontal cortex regions exhibit similar activation patterns as FC. Another fMRI study (body self-identification task) on 30 cisgenders and 30 transgenders found greater involvement of the limbic system in transgenders, who activated similar self- and body-processing neural systems aligned with their experienced gender and not with their birth-assigned sex (Majid et al., 2020).

#### Sexual Orientation

**Fig. 5:**
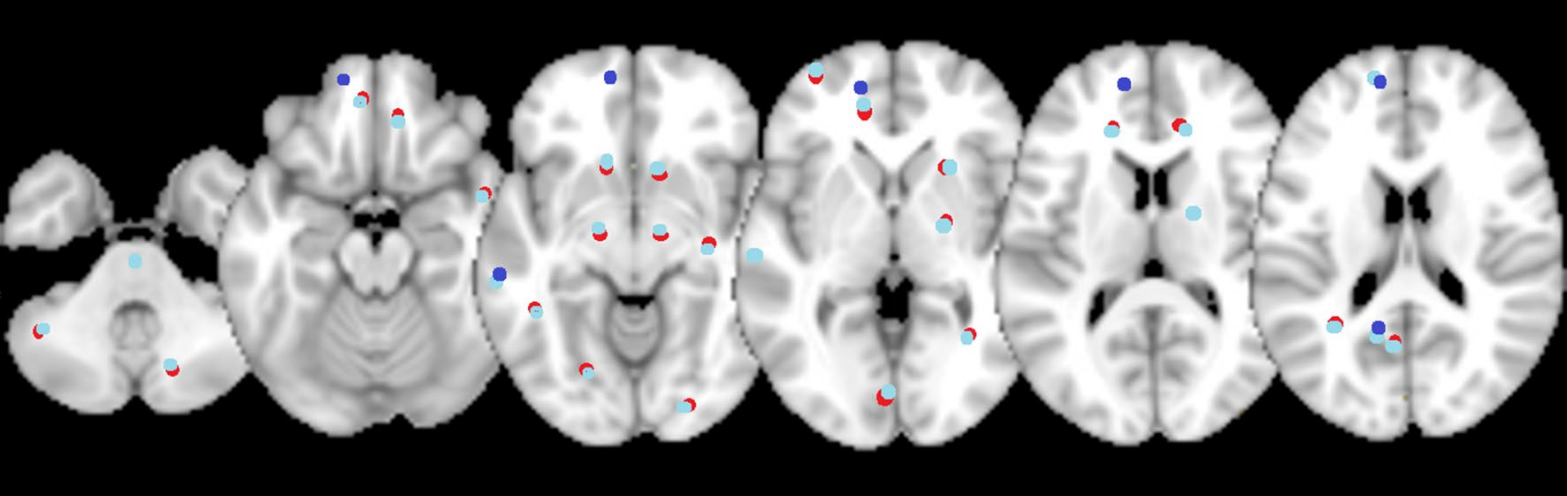
Six representative axial slices showing the foci resultant from the meta-analyses of stereotaxic coordinates where significant differences were found between groups with opposite sexual orientation (result from GingerAle 2.3.6; figure generated with micron.exe) (Red = Homosexual_vs_Heterosexual Natal Sex; cyan = Homosexual_vs_Heterosexual Opposite Sex; blue = Transgender_vs_Natal and Opposite Sex; indigo = Homosexual_vs_Heterosexual)

Eleven fMRI, 3 rs-fMRI, and 1 VBM studies published up to 2018 were analyzed. fMRI studies were conducted under visual stimulation (10) and an emotional judgment task (1). The 15 studies that conducted stereotaxic coordinates analysis involved 227 HoM, 94 HoW, 252 HeM, and 117 HeW. Figure 5 shows six representative slices with the foci resultant from the meta-analysis carried out using GingerAle 2.3.6 software (See Appendix 4 in Supplementary Materials for the labels of each foci and Table 6 for the number of foci related to different brain areas).

**Table 6:** Stereotaxic coordinates analysis on sexual orientation (number of foci related to different brain areas)

Homosexual_vs_Heterosexual Natal Sex		Homosexual_vs_Heterosexual Opposite Sex		Homosexual_vs_Heterosexual	
BA 19	10	BA 23	3	BA 18	5
BA 11	7	Coroide plexus	1	BA 23	4
BA 18	6	BA 17	1	Caudate	3
Caudate	5	BA 21	1	BA 17	3
Cingulate	5	BA 25	1	BA 19	3
Cerebellum	4	BA 41	1	BA 40	3
BA 7	4	BA 45	1	Cingulate	2
Optical radiation	3			Insula	2
BA 10	3			Thalamus	2
BA 17	3			BA 10	2
BA 40	3			BA 11	2
Amygdala	2			BA 24	2
Brain stem	2			BA 39	2
Insula	2			Brain stem	1
Striatum	2			Coroide plexus	1
Thalamus	2			Globus pallidus	1
BA 22	2			Lateral sulcus	1
BA 24	2			Optical radiation	1
BA 32	2			Periaqueductal	1
BA 39	2			Posterior cingulate	1
Anterior cingulate cortex	1			Putamen	1
Corpus callosum	1			Singular gyrus	1
Globus pallidus	1			Striatum	1
Hippocampus	1			BA 4	1
Paracentral lobule	1			BA 7	1
Periaqueductal	1			BA 21	1
Postcentral gyrus	1			BA 22	1
Posterior cingulate	1			BA 25	1
Precentral gyrus	1			BA 31	1
Putamen	1			BA 32	1
Temporal pole	1			BA 41	1
Temporo parietal junction	1			BA 45	1
BA 6	1			BA 47	1
BA 23	1				
BA 25	1				
BA 30	1				
BA 31	1				

BA, Brodmann area; Homosexual_vs_Heterosexual Natal Sex, HoM_vs_HeM + HoW_vs_HeW; Homosexual_vs_Heterosexual Opposite Sex, HoM_vs_HeW + HoW_vs_HeM

The meta-analysis “Homosexual_vs_Heterosexual Natal Sex” showed different activations in the visual area (BA 18 and 19). “Homosexual_vs_Heterosexual Opposite Sex” revealed differences in BA 23, which corresponds to the posterior cingular cortex, known to be involved in emotion, memory, meditation, and intrinsic control networks. Finally, the meta-analysis “Homosexual_vs_Heterosexual” showed that homosexual people’s brain activation differs more frequently in all these three areas.

From April 2018 to March 2021, two additional publications exploring sexual orientation using fMRI were identified. One study (Folkierska-Żukowska et al., 2020) considered within-group variations attributable to nonconforming behaviors and applied a mental rotation task to 90 individuals (mean age: 27.09 (23 HoM nonconforming), 26.6 (23 HoM conforming), 25.59 (22 HeM), 26.33 (23 HeW) yearls old). The study recalls that the term nonconforming refers to sex-atypical behaviors, interests, hobbies, activity levels, and play partner preferences, which have been “reliably associated with human nonheterosexuality” (Folkierska-Żukowska et al., 2020). This study found similarities in activation patterns from nonconforming HoM and HeW in right superior frontal gyrus, tight angular gyrus, right amygdala, parahippocampal gyrus, middle temporal gyrus and precuneus, which authors referred as “cross-sex shift”. Both conforming and nonconforming HoM had statistically significantly different levels of activations from HeW in left medial superior frontal gyrus and in the precentral/paracentral gyri. Patterns of activations in the angular and middle temporal gyri differed between conforming and non-conforming HoM highlighting heterogeneity in brain function within HoM. The other study (Afdile et al., 2019) applied a social grouping task involving 14 HoM and 15 HeM, and found significant group differences in areas of the medial prefrontal cortex, frontal pole, anterior cingulate cortex, right temporal parietal junction and bilateral superior frontal gyri.

### Metabolic Analysis

#### Gender Identity

Three PET and one SPECT studies were analyzed. They involved 25 FtM, 45 MtF, 41 FC, and 49 MC. A PET study investigated the hypothalamic network in 12 gynephilic (i.e., sexual preference for women) MtF, 12 gynephilic MC, and 12 androphilic (i.e., sexual preference for men) FC under smelling stimulation with steroids. Transgender individuals reported an intermediate hypothalamic pattern of activation between males and females, with prevalent feminine features (Berglund et al., 2008). Another PET study investigated serotonin transporter distribution in 14 MtF of different sexual orientations, 13 MC, and 9 FC with unspecified sexual orientation. While MC reported a rightward asymmetry in the midcingulate cortex, MtF and FC did not (Kranz et al., 2014a). Another PET study investigated serotonin transporter distribution in 19 MtF, 14 FtM, 24 MC, and 11 FC. ROIs investigated included insular cortex, cingulate cortex, amygdala, caudate, hippocampus, hypothalamus, putamen, and thalamus. Sexual orientation was not specified for any of them. Serotonin reuptake transporter non-displaceable binding potential (BPnd) was lower in amygdala, caudate, insular cortex, hippocampus, and putamen in FtM with respect to MC (Kranz et al., 2015). One SPECT study investigated regional cerebral blood flow (rCBF) in 11 gynephilic FtM and 9 androphilic FC. Transgender subjects reported an increase in rCBF in the right insula and a decrease in rCBF in the left anterior cingulate cortex (ACC) (Nawata et al., 2010). Taken together, these results seem to suggest that in the studies analyzed, transgender people not under hormonal treatment have certain brain metabolic features which tend to be slightly different from their natal sex and which are either similar to the opposite sex or intermediate between the two sexes.

#### Sexual Orientation

Four PET studies were analyzed. They involved 32 HoM, 24 HoW, 44 HeM, and 37 HeW. Hypothalamic activation under smelling stimulation with AND (i.e., progesterone derivative) and EST (i.e., estrogen-like steroid) was investigated in two studies (Berglund et al., 2006; Savic et al., 2005). Berglund et al. found a different preoptic hypothalamus activation between HoW and HeM with AND, while Savic et al. found a different preoptic and ventromedial hypothalamus activation between HoW and HeM with AND. Another study explored the brain activation in 8 HoM and 7 HeM in response to fluoxetine (i.e., selective serotonin reuptake inhibitor). With regards to the areas which are known to play a role in sexual behavior, HoM were reported to have a lower decrease in hypothalamic glucose metabolism than HeM. With regard to the areas which are not known to play a role in sexual behavior, HoM exhibited an increase of the glucose metabolism in the cingulate cortex, where HeM were reported to have a decrease; and in the prefrontal cortex, where no changes were reported for HeM (Kinnunen et al., 2004). Another study analyzed functional connectivity in 12 HoM, 12 HoW, 13 HeM, and 13 HeW. HoM and HeW exhibited more connections from the left amygdala with the contralateral amygdala, hypothalamus, subcallosum, and the anterior cingulate, while HoW and HeM exhibited more connections from the right amygdala with caudate and putamen (Savic & Lindström, 2008). Taken together, these results seem to suggest that homosexual individuals have some brain metabolic features that slightly differ from heterosexual individuals of their natal sex and others that are similar to heterosexuals of the opposite sex.

### Analysis of Bias

Appendix 5 and 6 show the risk of bias of the papers published up to 2018, calculated using the Quadas tool (see Supplementary Materials). Only 5/14 questions were applicable to our research. In all studies on both gender identity and sexual orientation, the samples were not representative of the population. Selection criteria were described clearly in only 31/51 papers (i.e., 16/30 on gender identity and 15/21 on sexual orientation). Texts were explanatory enough so as it can be replicated in 44/51 papers (i.e., 24/30 on gender identity and 20/21 on sexual orientation). Intermediate results were reported in 46/51 papers (i.e., 26/30 on gender identity and 20/21 on sexual orientation). Withdrawals (i.e., individuals who enrol a study and withdraw from it afterwards) from the studies included in this review were explained in all cases that referred it (i.e., 5/5 studies on gender identity and 3/3 studies on sexual orientation).

## Discussion

### Main Findings

The results from our systematic review and meta-analyses do not allow us to conclude on the specific brain phenotypes differential for each of the groups covered by this review. Although functional MRI studies (i.e., involving either fMRI or rs-MRI) on gender identity seem to indicate that fronto-parietal and cingulo-opercular brain regions are differentially relevant in transgenderism, a clear pattern accompanied by consistent structural changes is still to be found. Studies on gender identity with moderate-to-larger samples which included individuals with different sexual orientation in their control groups (Baldinger-Melich et al., 2020; Manzouri & Savic, 2019), exposed the complexities underlying both gender identity and sexual orientation. The data extracted may suggest that before hormonal treatment the majority of transgenders’ brain features covered by the studies reviewed could be similar to those of their natal sex, but certainly some brain parameters differ resembling those of their experienced gender. Also, although homosexual’s neuroanatomy, neurophysiology, and neurometabolism may tend to resemble those of heterosexual individuals of their same sex, some brain features differ and are similar to those of heterosexual individuals of the opposite sex in some of the studies analyzed.

The compilation of the data from the studies included shows neural differences between the groups studied. However, brain functions are mediated by different brain areas and their interactions, rather than by single structures. The correlation or association between a certain brain function, volumetric change or activation, with a certain activity and/or behavior does not establish whether (or not) that structure/function is causally important for that activity/behavior (Koob et al., 2013; Maney, 2016). It merely shows a possible involvement or apparent trend. Complex human behaviors (and few simple behaviors) cannot be entirely explained by phenomena occurring only in a single brain region. Therefore, the idea that brain sexual differences cause behavioral sexual differences, rather than being an assumption, still constitutes a hypothesis to verify.

Studies on cisgender and heterosexual samples have reported sex differences in brain anatomy on a global scale, regarding absolute volumes (Kurth et al., 2016). Studies have also reported sexual dimorphism in the relative sizes and shapes of regional brain structures, with the direction of the sex effect varying between regions, including the Broca’s region (Kurth et al., 2016), corpus callosum (Prendergast et al., 2015), amygdala and hippocampus (Giedd et al., 1996). These findings reflect on the selectivity of the brain regions analyzed by the studies included in this review. However, research investigating differences at the level of regional tissue volumes is highly contradictory. A large study that analyzed MRI data of 1400 cisgender heterosexual individuals from four different datasets (Joel et al., 2015) found substantial overlap in the distribution of anatomical traits between males and females in all brain regions and connections examined, undermining attempts to clearly distinguish between “male” and “female” forms of specific brain features. They arrived at the idea that human brains cannot in fact, be distinctly categorized into two distinct classes but rather, that male and female brains are comprised of “unique mosaics” of features, some of which are more common in one sex than the other and some that are common in both.

Some authors refer to an early programming of gender and sexual inclination driven by sexual differentiation in the brain, proposing that the latter influences the development of the brain areas modulating body perception (i.e., related to gender identity) or sexual arousal (i.e., related to sexual orientation) (Burke et al., 2017; Manzouri & Savic, 2019). Others underline the interaction between brain, culture and behavior, arguing that structural and functional brain changes in transgender individuals may be consequence of culture and behavior (Mohammadi & Khalegi, 2018). The etiology and drivers of differences in gender identity and sexual orientation is out of the scope of this review, and caution must be exercised to drive conclusions from the neuroscience literature alone, as human behavior, ultimately, is not reducible to biological nor to cultural factors, but is a consequence of their interaction. As such, human sexuality is a multilevel complex, and the challenge is to investigate how biological, historical and cultural elements interact with each other.

#### Regions of Interest Analysis

The lack of data did not allow us to meta-analyze the information obtained from the studies that conducted ROI analyses. From extracting and summarizing all the information available, differences were found between cisgender and transgender people in white matter microstructure, volumetric analyses, cortical thickness, and corpus callosum shape. Differences between heterosexual and homosexual people were found in cortical thickness, subcortical volumes, and cerebral hemisphere, but not in white matter tracts. The studies included, in the rest of the ROIs analyzed, either did not find significant differences between cisgender and transgender brains nor between heterosexual and homosexual; or found significant differences just between transgenders and opposite sex cisgenders, and between homosexuals and opposite sex heterosexuals (see Tables 3 and 4). Our findings on gender identity are consistent with previous studies that also attempted to summarize the literature findings on this topic, according to which gross morphology in transgenders is more similar to cisgender people of their natal sex than to cisgender people of their experienced gender (Guillamon et al., 2016; Kreukels & Guillamon, 2016; Mueller et al., 2017; Smith et al., 2015), even though white matter microstructure (Kreukels & Guillamon, 2016; Mueller et al., 2017; Smith et al., 2015), cortical thickness (Guillamon et al., 2016; Smith et al., 2015), and subcortical volumes (Mueller et al., 2017) may deviate from the biological sex towards values of experienced gender.

#### Stereotaxic Coordinates Analysis

Occipital brain regions, involved in visual processing, are the ones that most frequently were found to have a different activation in cisgenders compared to transgenders, followed by some fronto-temporal foci. This is not surprising given that, in general, most fMRI studies involved in both analyses involved visual stimulation. In addition, specifically the BA 23 had different activations for heterosexuals with respect to homosexuals. Our meta-analysis found different brain activations between different groups scattered across the whole brain, but overall with low frequency (see Tables 5 and 6). Our results on gender identity are consistent with some of the previous studies mentioned above, according to which in certain brain areas transgenders’ activation is closer to those of their experienced gender (Guillamon et al., 2016; Smith et al., 2015). While there is still concensus that a clear picture has yet to emerge (Mueller et al., 2017), recent advances in artificial intelligence confirm the observations above, by indicating that some fronto-parietal and cingulo-opercular areas may be of relevance for predicting hormonal therapy outcomes (Moody et al., 2021).

#### Metabolic Analysis

In transgenders and homosexuals, some metabolic features seem to differ slightly from cisgenders of their natal sex and from heterosexuals of their natal sex respectively. However, given the reduced number of studies included that conducted these analyses, these findings cannot be generalized. This is in line with what the scientific literature on gender identity up to date has concluded in this respect (Smith et al., 2015).

### Strengths and Limitations

To the best of our knowledge, this is the first systematic review and meta-analysis of the neuroimaging literature on structural, functional, and metabolic differences as a function of both gender identity (before the hormonal treatment) and sexual orientation. In addition, we carefully extracted and processed all data from all studies considered for meta-analyses and made them publicly available to facilitate further research in this important area.

Several limitations regarding the small sample size of the meta-analysis and the heterogeneity of the investigations must be acknowledged. The analyses of our systematic search up to 2018 included 51 studies (i.e., 30 on gender identity and 21 on sexual orientation) all with relatively small samples, conducted with different neuroimaging techniques (1 SPECT, 3 PET, 6 fMRI, 8 rs-fMRI, and 13 MRI on gender identity; 4 PET, 5 MRI, 3 rs-fMRI, and 11 fMRI on sexual orientation). Different studies conducted with MRI investigated different brain structures (cortex, subcortical volumes, white matter, CSF, and ventricles in gender identity; cortex, subcortical volumes, and white matter in sexual orientation). fMRI was conducted under different stimulations (1 smelling, 1 vocal stimulation, 1 mental rotation task, 1 verbal fluency test, and 2 visual in gender identity; 10 visual stimulation and 1 emotional judgment task in sexual orientation). Metabolic analysis investigated different brain areas (hypothalamic network, serotonin transport distribution in different ROI, and rCBF in gender identity studies; hypothalamic activation and functional connectivity in sexual orientation studies) using different neuroimaging techniques (PET and SPECT in gender identity research; PET in sexual orientation research). As a result, it was not possible to meta-analyze the results from all studies that fit our inclusion/exclusion criteria, and the main contribution of our work, therefore, is limited to the scientific compilation and synthesis of the data available. An update on the primary search conducted in one database, added 12 papers to the analyses which, although enriched the data presented, was rather confirmatory of our main findings and added heterogeneity to the results.

Moreover, some studies had some limitations regarding the presentation of their data. First, some studies did not report statistical parameters and just reported whether or not there were significant differences between cisgenders and transgenders and between heterosexuals and homosexuals. Second, other studies reported statistical parameters only in case of significant differences between groups, and omitted reporting negative results (i.e., when no differences were found) (gender identity investigation: Burke et al., 2014; Kranz et al., 2014b, 2015; Ku et al., 2013; Lin et al., 2014; Luders et al., 2009; Nota et al., 2017; Pol et al., 2006; Santarnecchi et al., 2012; Soleman et al., 2013; Spies et al., 2016; Yokota et al., 2005; Zubiaurre-Elorza et al., 2013; sexual orientation investigation: Hu et al., 2008; Ponseti et al., 2007; Savic and Lindström, 2008; Sylva, 2013; Zeki & Romaya, 2010; for more detailed information, please see analysis of bias in Appendix 5 and 6). A complete presentation of scientific data, including negative results, is important to precisely evaluate scientific investigations on a certain topic (Matosin et al., 2014).

Information on the biological sex of the studies’ participants is part of the scientific data we collected and made available. The data presented show MtF and FtM transgender individuals do not have mirror images of brain differences. However, the heterogeneity of the design of the studies involved, despite enriching the scope of this review, due to the limited number of studies included and their sample sizes, made it impossible to draw conclusions on specific biological sex differences for the groups covered in this review. For example, some papers compared MtF with MC, others MtF with FC, others FtM with MC, and others FtM with FC. The studies included in this review on transgenderism did not provide information on early-onset or late-onset transgenderism. Therefore, analysis and information of this important point is lacking.

Finally, as Guillamon et al. (2016) noted, some studies conducted on gender identity did not report the sexual orientation of the individuals that constituted their sample. Gender identity and sexual orientation are conceptually different, i.e., both cisgender and transgender people are either heterosexual or homosexual (Burke et al., 2017; Moser, 2010), and there are more gender identities other than cis-/transgender(ism) (such as genderqueer or non-binary) and other sexual orientations other than hetero-/homosexual(ism) (such as bi-, pan-, and asexual). Sexual orientation could be associated with brain structural specific features regardless and independently from gender identity as some recent studies suggest (Baldinger-Melich et al., 2020; Manzouri & Savic, 2019). Thus, meaning that the structural, functional, and metabolic variations found in homosexual transgenders with respect to heterosexual cisgenders may be related to their sexual orientation rather than to their gender identity (Blanchard et al., 1987). A recent study identified brain regions where both sexual orientation and gender identity seemingly interact (Wang, Han, et al., 2020).

### Conclusions and Future Directions

Over the past few years, the neuroimaging investigation on human sexuality has increased and several studies on gender identity and sexual orientation comparing cisgenders vs. transgenders and heterosexuals vs. homosexuals have been conducted.

This review explored structural, functional, and metabolic features of cisgenders compared to transgenders before hormonal treatment and heterosexuals compared with homosexuals. Results suggest that, although the majority of neuroanatomical, neurophysiological, and neurometabolic features in transgenders resemble those of their natal sex rather than those of their experienced gender, and in homosexuals these resemble those of their same-sex heterosexual population rather than their opposite sex heterosexual population, in the gender identity investigation, in MtF it was possible to find traits which are “feminine and demasculinized” and in FtM it was possible to find traits which are “masculine and defeminized” (Kreukels & Guillamon, 2016). The same could be said with regard to the investigation on sexual orientation, where some brain features in the homosexual population from the studies reviewed resembled those of the heterosexual population of their opposite sex. Due to conflicting results, it was, however, not possible to identify specific brain features which consistently differ between cisgender and transgender nor between heterosexual and homosexual groups. Very small brain changes, to date undetectable using the current neuroimaging tools, may affect behavior. The small number of studies, the small sample size of each study, the heterogeneity of investigations, the lack of negative results reported by some studies, and the fact that some studies did not report the sexual orientation of the individuals that composed their sample did not allow drawing general conclusions. Moreover, as the samples of the publications involved are not representative of the population analyzed, caution should be taken in the interpretation of the results of this review.

To overcome the limitations mentioned above, future studies should: (1) keep investigating brain areas which are sexually dimorphic (e.g., hypothalamus, hippocampus, caudate, corpus callosum, and serotonin transport) and brain areas involved in processing own-body perception (e.g., parietal, frontal, insular cortex, and its connections with thalamus and putamen) and sexual stimuli and arousal (e.g., hypothalamus and ventral striatum); (2) conduct Metabolic Analysis along with structural and functional to increase the number of data available; (3) report both positive and negative results to conduct an unbiased statistical analysis; (4) report sexual orientation of individuals that comprise the sample size in studies on gender identity; (5) increase the sample size and expand the age range of the sample; (6) differentiate with respect to early- or late-onset transgenderism to reach a better understanding of the biological features underlying them. Future reviews in the topic should extend the inclusion criteria to distinguish between pre- vs. post-pubertal and pre- vs. post-hormonal treatment, as well as include other advanced neuroimaging modalities such as magnetic resonance spectroscopy, and dynamic sequence acquisitions to increase the value and scope of the present report.

## Supplementary Information

Below is the link to the electronic supplementary material.

Supplementary file 1 (DOCX 59 KB)

Supplementary file 2 (XLSX 65 KB)

Supplementary file 3 (XLSX 63 KB)
