# Supplementary material for: Structural, Functional, and Metabolic Brain Differences as a Function of Gender Identity or Sexual Orientation: A Systematic Review of the Human Neuroimaging Literature
Source: Arch Sex Behav. 2021 May 6;50(8):3329–52. doi: 10.1007/s10508-021-02005-9 (PMC8604863; doi:10.1007/s10508-021-02005-9)
Supplement: Supplementary file 1 — (DOCX 59 KB) [file 10508_2021_2005_MOESM1_ESM.docx]

**Structural, Functional, and Metabolic Brain Differences as a Function of Gender Identity or Sexual Orientation: A Systematic Review of the Human Neuroimaging Literature**

**SUPPLEMENTARY MATERIAL**

**Appendix 1 – Search Strategy_Gender Identity Research**

Search Strategy_Embase

1. exp gender dysphoria/

2. gender dysphoria.tw.

3. gender identity disorder.tw.

4. gender disorder.tw.

5. gid.tw.

6. exp transgender/

7. exp female to male transgender/

8. exp male to female transgender/

9. transgender.tw.

10. exp transgenderism/

11. transgenderism.tw.

12. transgender$.mp. [mp=title, abstract, heading word, drug trade name, original title, device manufacturer, drug manufacturer, device trade name, keyword, floating subheading word]

13. exp transsexuality/

14. transsexuality.tw.

15. transsexualism.tw.

16. transsexual$.mp. [mp=title, abstract, heading word, drug trade name, original title, device manufacturer, drug manufacturer, device trade name, keyword, floating subheading word]

17. exp gender identity/

18. gender identity.tw.

19. 1 or 2 or 3 or 4 or 5 or 6 or 7 or 8 or 9 or 10 or 11 or 12 or 13 or 14 or 15 or 16 or 17 or 18

20. exp brain/

21. brain.tw.

22. 20 or 21

23. exp computer assisted tomography/

24. computer assisted tomography.tw.

25. computer axial tomography.tw.

26. cat.tw.

27. computer tomography.tw.

28. ct.tw.

29. exp nuclear magnetic resonance/

30. nuclear magnetic resonance.tw.

31. nmr.tw.

32. nuclear magnet resonance imaging.tw.

33. nmr imaging.tw.

34. magnetic resonance imaging.tw.

35. nm imaging.tw.

36. mri.tw.

37. mri scan.tw.

38. magnetic resonance.tw.

39. mr.tw.

40. mr scan.tw.

41. exp diffusion tensor imaging/

42. diffusion tensor imaging.tw.

43. dti.tw.

44. exp functional magnetic resonance imaging/

45. functional magnetic resonance imaging.tw.

46. exp BOLD signal/

47. bold.tw.

48. resting state imaging.tw.

49. rs-fmri.tw.

50. exp single photon emission computed tomography/

51. single photon emission computer tomography.tw.

52. spect.tw.

53. exp CT-SPECT scanner/

54. positron emission tomography.tw.

55. exp positron emission tomography/

56. pet.tw.

57. exp PET-CT scanner/

58. exp voxel based morphometry/

59. voxel based morphometry.tw.

60. vbm.tw.

61. exp diffusion weighted imaging/

62. diffusion weighted imaging.tw.

63. dwi.tw.

64. 23 or 24 or 25 or 26 or 27 or 28 or 29 or 30 or 31 or 32 or 33 or 34 or 35 or 36 or 37 or 38 or 39 or 40 or 41 or 42 or 43 or 44 or 45 or 46 or 47 or 48 or 49 or 50 or 51 or 52 or 53 or 54 or 55 or 56 or 57 or 58 or 59 or 60 or 61 or 62 or 63

65. 19 and 22 and 64

Search Strategy_Medline

1. exp Gender Dysphoria/

2. gender dysphoria.tw.

3. exp Gender Identity/

4. gender identity.tw.

5. gender identity disorder.tw.

6. gender disorder.tw.

7. exp Transgender Persons/

8. transgender.tw.

9. transgenderism.tw.

10. transgender$.mp. [mp=title, abstract, original title, name of substance word, subject heading word, keyword heading word, protocol supplementary concept word, rare disease supplementary concept word, unique identifier, synonyms]

11. exp TRANSSEXUALISM/

12. transsexualism.tw.

13. transsexuality.tw.

14. transsexual$.mp. [mp=title, abstract, original title, name of substance word, subject heading word, keyword heading word, protocol supplementary concept word, rare disease supplementary concept word, unique identifier, synonyms]

15. 1 or 2 or 3 or 4 or 5 or 6 or 7 or 8 or 9 or 10 or 11 or 12 or 13 or 14

16. exp BRAIN/

17. brain.tw.

18. 16 or 17

19. exp TOMOGRAPHY, X-RAY/

20. computer assisted tomography.tw.

21. computer axial tomography.tw.

22. cat.tw.

23. computer tomography.tw.

24. ct.tw.

25. nuclear magnetic resonance.tw.

26. nmr.tw.

27. nuclear magnetic resonance imaging.tw.

28. nmr imaging.tw.

29. exp Magnetic Resonance Imaging/

30. magnetic resonance imaging.tw.

31. mr imaging.tw.

32. mri scan.tw.

33. mri.tw.

34. magnetic resonance.tw.

35. mr.tw.

36. mr scan.tw.

37. exp Diffusion Tensor Imaging/

38. diffusion tensor imaging.tw.

39. dti.tw.

40. functional magnetic resonance imaging.tw.

41. fmri.tw.

42. bold.tw.

43. resting state imaging.tw.

44. rs-fmri.tw.

45. exp TOMOGRAPHY, EMISSION-COMPUTED, SINGLE-PHOTON/

46. single photon emission computer tomography.tw.

47. spect.tw.

48. exp Positron-Emission Tomography/

49. pet.tw.

50. positron emission tomography.tw.

51. exp Image Processing, Computer-Assisted/

52. voxel based morphometry.tw.

53. vbm.tw.

54. exp Diffusion Magnetic Resonance Imaging/

55. diffusion weighted imaging.tw.

56. dwi.tw.

57. 19 or 20 or 21 or 22 or 23 or 24 or 25 or 26 or 27 or 28 or 29 or 30 or 31 or 32 or 33 or 34 or 35 or 36 or 37 or 38 or 39 or 40 or 41 or 42 or 43 or 44 or 45 or 46 or 47 or 48 or 49 or 50 or 51 or 52 or 53 or 54 or 55 or 56

58. 15 and 18 and 57

Search Strategy_PsycINFO

1. exp Gender Identity Disorder/

2. gender identity disorder.tw.

3. gender disorder.tw.

4. gender dysphoria.tw.

5. exp Gender Identity/

6. gender identity.tw.

7. exp TRANSGENDER/

8. transgender.tw.

9. transgenderism.tw.

10. transgender$.mp. [mp=title, abstract, heading word, table of contents, key concepts, original title, tests & measures]

11. exp TRANSSEXUALISM/

12. transsexualism.tw.

13. transsexuality.tw.

14. transsexual$.mp. [mp=title, abstract, heading word, table of contents, key concepts, original title, tests & measures]

15. 1 or 2 or 3 or 4 or 5 or 6 or 7 or 8 or 9 or 10 or 11 or 12 or 13 or 14

16. exp BRAIN/

17. brain.tw.

18. 16 or 17

19. computer assisted tomography.tw.

20. computer axial tomography.tw.

21. cat.tw.

22. computer tomography.tw.

23. ct.tw.

24. exp Magnetic Resonance Imaging/

25. magnetic resonance imaging.tw.

26. mr imaging.tw.

27. mri.tw.

28. mri scan.tw.

29. nuclear magnetic resonance.tw.

30. nmr.tw.

31. nuclear magnetic resonance imaging.tw.

32. nmr imaging.tw.

33. magnetic resonance.tw.

34. mr.tw.

35. mr scan.tw.

36. exp Diffusion Tensor Imaging/

37. diffusion tensor imaging.tw.

38. dti.tw.

39. exp Functional Magnetic Resonance Imaging/

40. functional magnetic resonance imaging.tw.

41. fmri.tw.

42. bold.tw.

43. resting state imaging.tw.

44. rs-fmri.tw.

45. exp Single Photon Emission Computed Tomography/

46. single photon emission computer tomography.tw.

47. spect.tw.

48. exp Positron Emission Tomography/

49. positron emission tomography.tw.

50. pet.tw.

51. vbm.tw.

52. diffusion weighted imaging.tw.

53. dwi.tw.

54. 19 or 20 or 21 or 22 or 23 or 24 or 25 or 26 or 27 or 28 or 29 or 30 or 31 or 32 or 33 or 34 or 35 or 36 or 37 or 38 or 39 or 40 or 41 or 42 or 43 or 44 or 45 or 46 or 47 or 48 or 49 or 50 or 51 or 52 or 53

55. 15 and 18 and 54

**Appendix 2 – Search Strategy_Sexual Orientation Research**

Search Strategy_Embase

1. exp homosexuality/

2. homosexuality.tw.

3. homosex$.mp. [mp=title, abstract, heading word, drug trade name, original title, device manufacturer, drug manufacturer, device trade name, keyword, floating subheading word, candidate term word]

4. exp homosexual male/

5. homosexual male.tw.

6. exp homosexual female/

7. homosexual female.tw.

8. gay.tw.

9. lesbian.tw.

10. 1 or 2 or 3 or 4 or 5 or 6 or 7 or 8 or 9

11. exp brain/

12. brain.tw.

13. 11 or 12

14. exp nuclear magnetic resonance/

15. nuclear magnetic resonance.tw.

16. nmr.tw.

17. nuclear magnetic resonance imaging.tw.

18. nmr imaging.tw.

19. magnetic resonance imaging.tw.

20. mri.tw.

21. magnetic resonance.tw.

22. mr.tw.

23. diffusion tensor imaging.tw.

24. dti.tw.

25. exp functional magnetic resonance imaging/

26. functional magnetic resonance imaging.tw.

27. exp BOLD signal/

28. bold.tw.

29. resting state imaging.tw.

30. rs-fMRI.tw.

31. exp single photon emission computed tomography/

32. single photon emission computed tomography.tw.

33. spect.tw.

34. exp CT-SPECT scanner/

35. exp positron emission tomography/

36. positron emission tomography.tw.

37. pet.tw.

38. exp PET-CT scanner/

39. exp voxel based morphometry/

40. voxel based morphometry.tw.

41. vbm.tw.

42. exp diffusion weighted imaging/

43. dwi.tw.

44. 14 or 15 or 16 or 17 or 18 or 19 or 20 or 21 or 22 or 23 or 24 or 25 or 26 or 27 or 28 or 29 or 30 or 31 or 32 or 33 or 34 or 35 or 36 or 37 or 38 or 39 or 40 or 41 or 42 or 43

45. 10 and 13 and 44

46. limit 45 to (article or article in press or books or chapter or conference paper or reports)

Search Strategy_Medline

1. exp HOMOSEXUALITY/

2. homosexuality.tw.

3. homosex$.mp. [mp=title, abstract, original title, name of substance word, subject heading word, keyword heading word, protocol supplementary concept word, rare disease supplementary concept word, unique identifier, synonyms]

4. exp HOMOSEXUALITY, FEMALE/

5. homosexuality female.tw.

6. exp HOMOSEXUALITY, MALE/

7. homosexuality male.tw.

8. gay.tw.

9. lesbian.tw.

10. 1 or 2 or 3 or 4 or 5 or 6 or 7 or 8 or 9

11. exp BRAIN/

12. brain.tw.

13. 11 or 12

14. nuclear magnetic resonance.tw.

15. nmr.tw.

16. nuclear magnetic resonance imaging.tw.

17. nmr imaging.tw.

18. exp Magnetic Resonance Imaging/

19. magnetic resonance imaging.tw.

20. mri.tw.

21. magnetic resonance.tw.

22. mr.tw.

23. mr scan.tw.

24. diffusion tensor imaging.tw.

25. dti.tw.

26. functional magnetic resonance imaging.tw.

27. fmri.tw.

28. bold.tw.

29. resting state imaging.tw.

30. rs-fmri.tw.

31. exp TOMOGRAPHY, EMISSION-COMPUTED, SINGLE-PHOTON/

32. single photon emission computed tomography.tw.

33. spect.tw.

34. exp POSITRON-EMISSION TOMOGRAPHY/

35. pet.tw.

36. positron emission tomography.tw.

37. voxel based morphometry.tw.

38. vbm.tw.

39. exp Diffusion Magnetic Resonance Imaging/

40. diffusion weighted imaging.tw.

41. dwi.tw.

42. 14 or 15 or 16 or 17 or 18 or 19 or 20 or 21 or 22 or 23 or 24 or 25 or 26 or 27 or 28 or 29 or 30 or 31 or 32 or 33 or 34 or 35 or 36 or 37 or 38 or 39 or 40 or 41

43. 10 and 13 and 42

44. limit 43 to (adaptive clinical trial or case reports or classical article or clinical study or clinical trial, all or comparative study or evaluation studies or interactive tutorial or journal article or letter or multicenter study or observational study or technical report or validation studies)

Search Strategy_PsycINFO

1. exp HOMOSEXUALITY/

2. homosexuality.tw.

3. homosex$.mp. [mp=title, abstract, heading word, table of contents, key concepts, original title, tests & measures]

4. exp Male Homosexuality/

5. male homosexuality.tw.

6. gay.tw.

7. exp Lesbianism/

8. lesbian.tw.

9. female homosexuality.tw.

10. 1 or 2 or 3 or 4 or 5 or 6 or 7 or 8 or 9

11. exp BRAIN/

12. brain.tw.

13. 11 or 12

14. exp Magnetic Resonance Imaging/

15. magnetic resonance imaging.tw.

16. mri.tw.

17. nuclear magnetic resonance.tw.

18. nmr.tw.

19. nuclear magnetic resonance imaging.tw.

20. nmr imaging.tw.

21. magnetic resonance.tw.

22. mr.tw.

23. exp Diffusion Tensor Imaging/

24. diffusione tensor imaging.tw.

25. dti.tw.

26. exp Functional Magnetic Resonance Imaging/

27. functional magnetic resonance imaging.tw.

28. fmri.tw.

29. bold.tw.

30. resting state imaging.tw.

31. rs-fmri.tw.

32. exp Single Photon Emission Computed Tomography/

33. single photon emission computed tomography.tw.

34. spect.tw.

35. exp Positron Emission Tomography/

36. positron emission tomography.tw.

37. pet.tw.

38. voxel based morphometry.tw.

39. vbm.tw.

40. diffusion weighted imaging.tw.

41. dwi.tw.

42. 14 or 15 or 16 or 17 or 18 or 19 or 20 or 21 or 22 or 23 or 24 or 25 or 26 or 27 or 28 or 29 or 30 or 31 or 32 or 33 or 34 or 35 or 36 or 37 or 38 or 39 or 40 or 41

43. 10 and 13 and 42

44. limit 43 to (chapter or journal article or letter)

**Appendix 3 – Stereotaxis Coordinates Analysis_Gender Identity Research**

Transgender_vs_Assigned Sex at Birth

//Burke et al, 2014

//Subjects=39

2 -14 -2 Hypothalamus R

0 -10 0 Hypothalamus

-4 -6 -4 Hypothalamus L

0 -10 -12 Hypothalamus

//Junger et al, 2014

//Subjects=37

-12 -37 -11 Lingual Gyrus L

9 -97 22 Cuneus

-9 -97 -8 Lingual Gyrus L

45 11 1 Insula

18 -94 -5 Calcarine Gyrus R

51 41 7 Area Triangularis

//Schöning et al, 2010

//Subjects=22

-42 -26 30 Parietal Lobe L

-38 -54 64 Parietal Lobe L

-6 4 58 Frontal Gyrus L

42 6 14 Insular Cortex R

-24 18 32 Frontal Gyrus L

24 28 60 Frontal Gyrus R

10 44 38 Frontal Gyrus R

30 38 -2 Frontal Gyrus R

30 18 42 Frontal Gyrus R

-52 -60 12 Temporal Gyrus L

-36 -56 6 Temporal Gyrus L

-48 -16 -16 Occipital Gyrus L

12 6 16 Caudate R

6 -64 38 Precuneus R

-10 -62 -26 Cerebellum L

//Gizewski et al, 2009

// Subjects=24

-26 -3 -18 Amygdala L

30 -18 -15 Amygdala R

-6 -21 6 Thalamus L

-6 42 -15 Orbitofrontal L

12 27 -21 Orbitofrontal R

-42 -3 -12 Insular L

51 -11 11 Insular R

//Burke et al, 2014

//Subjects=36

-6 -10 -6 Hypothalamus L

3 -16 -9 Midbrain R

-2 -16 -6 Midbrain L

-6 -10 -6 Hypothalamus L

//Burke et al, 2016

//Subjects=42

57 6 24 Frontal Cortex R

//Clemens et al, 2014

//Subjects=36

54 -16 -18 Inferior Temporal Gyrus

16 -62 18 Calcarine Gyrus

//Feusner et al, 2017

//Subjects=54

6 54 0 Anterior Cingulate Cortex R

-8 38 20 Anterior Cingulate Cortex L

12 -52 28 Right Precuneus

-2 -30 34 Posterior Cingulate Cortex L

24 -84 12 Occipital Cortex R

20 -94 -6 Occipital Cortex R

-14 -80 -12 Occipital Cortex L

-10 -76 18 Occipital Cortex L

-6 -72 6 Occipital Cortex L

//Manzouri et al, 2017

//Subjects=62

-10 -42 -5 Fusiform L

20 -66 -2 Fusiform R

-8 -56 42 Precuneus L

12 -2 14 Thalamus R

6 -66 6 Anterior Cingulate Cortex R

32 -88 6 Precuneus R

-22 26 22 Frontal Gyrus L

24 52 14 Frontal Gyrus R

//Savic and Arver, 2011

//Subjects=48

49 -40 18 Temporal Gyrus R

39 15 -16 Insular Cortex R

13 -28 5 Thalamus R

13 45 32 Frontal WM R

//Santarnecchi et al, 2012

//Subjects=26

-33 3 35 Lingual Gyrus L

Transgender_vs_Opposite Sex

//Junger et al, 2014

//Subjects=36

6 -91 28 Cuneus

6 59 16 Medial Prefrontal Cortex R

-15 53 7 Medial Prefrontal Cortex L

12 -40 -29 Cerebellum R

48 -43 16 Superior Temporal Gyrus R

-6 -37 55 Precuneus L

0 -16 -2 Thalamus

21 -25 58 Precentral Gyrus R

//Clemens et al, 2014

//Subjects=35

-6 32 50 Prefrontal Cortex L

6 2 44 Midcingulate Cortex R

-4 -20 28 Posterior Cingulate Cortex L

52 -26 28 Inferior Parietal Gyrus R

6 -22 2 Thalamus R

//Feusner et al, 2017

//Subjects=54

0 44 4 Anterior Cingulate Cortex

-2 -66 30 Precuneus L

-2 -26 26 Posterior Cingulate Cortex L

38 -88 -16 Occipital Cortex R

//Manzouri et al, 2017

//Subjects=62

36 -14 18 Gyrus R

28 -27 -8 Gyrus R

-14 -45 0 Fusiform L

6 -3 18 Hypothalamus R

15 -42 -2 Fusiform R

45 -46 -22 Parietal Cortex R

0 -91 12 Cuneus R

-6 -56 48 Precuneus L

26 14 4 Thalamus R

45 20 -6 Insular Cortex R

2 58 6 Anterior Cingulate Cortex R

14 -76 -12 Frontal Gyrus L

-28 16 26 Frontal Gyrus R

//Savic and Arver, 2011

//Subjects=48

41 16 -12 Insular Cortex R

52 -33 16 Temporal Gyrus R

10 -89 -22 Lingual Gyrus R

-30 -90 -24 Cerebellum L

-1 -13 5 Thalamus L

-39 -20 44 Central Gyrus L

-4 -11 49 Central Gyrus L

//Santarnecchi et al, 2012

//Subjects=26

-6 -54 -22 Cerebellum L

//Hoekzema et al, 2015

//Subjects=98

-42 -78 -18 Fusiform L

//Hoekzema et al, 2015

//Subjects=89

18 57 33 Superior Medial R

6 59 37 Cerebellum R

-15 -48 -59 Cerebellum L

Transgender_vs_Assigned at Birth & Opposite Sex

//Simon et al, 2013

//Subjects=35

34.5 -72 -10.5 Occipital Lobe R

54 -75 -4.5 Temporal Gyrus R

//Ku et al, 2013

//Subjects=46

-8 22 34 Anterior Cingulate Cortex

-8 40 18 Anterior Cingulate Cortex

-8 22 34 Anterior Cingulate Cortex

-8 40 18 Anterior Cingulate Cortex

**Appendix 4 – Stereotaxic Coordinates Analysis_Sexual Orientation Research**

Homosexual_vs_Heterosexual Assigned Sex at Birth

//Manzouri and Savic, 2018

//Subjects=70

-1 50 17 Anterior Cingulate Cortex

-6 -50 3 Precuneus

//Ponseti et al, 2007

//Subjects=40

-32 1 -35 Perirhinal Cortex L

-63 3 36 Ventral Premotor Cortex L

-10 -40 -55 Cerebellum L

26 -50 -59 Cerebellum R

34 3 -36 Perirhinal Cortex R

//Hu et al, 2008

//Subjects=20

-42 -68 48 Angular Gyrus L

-8 0 24 Caudate L

14 0 6 Pallidum R

2 -80 -2 Lingual Gyrus L

6 -70 2 Lingual Gyrus L

20 -36 -2 Hippocampus R

16 -36 -10 Parahippocampus R

//Hu et al, 2011

//Subjects=28

9 3 -15 Subcallosum Gyrus R

-30 -60 -21 Cerebellum L

12 48 0 Anterior Cingulate R

9 -42 9 Corpus Callosum R

12 -51 6 Posterior Cingulate R

0 -21 -30 Pons L

-30 -78 -6 Occipital Lobe L

12 -48 -6 Cerebellum R

21 -54 2 Subcallosum Gyrus R

6 3 -15 Subcallosum Gyrus R

0 -54 -42 Cerebellum L

33 -3 42 Frontal Lobe R

42 0 39 Precentral Gyrus R

15 -21 45 Frontal Lobe R

39 -57 27 Superior Temporal Gyrus R

33 -63 33 Angular Gyrus R

51 -6 -18 Middle Temporal Gyrus R

-45 -45 -9 Temporal Lobe L

24 -87 -9 Lingual Gyrus R

-12 42 0 Anterior Cingulate L

-18 51 6 Medial Frontal Gyrus L

18 -51 -36 Cerebellum R

-12 0 27 Cingulate Gyrus L

15 -72 -39 Cerebellum R

3 -45 15 Posterior Cingulate R

3 -48 69 Posterior Gyrus R

3 -99 3 Cuneus R

-42 -54 -39 Cerebellum L

-39 -51 -30 Cerebellum L

-24 33 -21 Inferior Frontal Gyrus L

-33 -72 -12 Middle Occipital Gyrus L

-18 36 12 Frontal Lobe L

9 36 15 Anterior Cingulate R

15 48 15 Medial Frontal Gyrus R

36 -54 -12 Fusiform Gyrus R

-6 -69 33 Precuneus L

//Hu et al, 2013

//Subjects=52

-6 24 -21 Rectal Gyrus L

-15 -12 -9 Midbrain L

12 -12 -9 Midbrain R

-33 -51 18 Temporal Lobe L

18 -3 12 Extra-Nuclear WM R

-42 -78 -6 Inferior Occipital Gyrus L

39 -90 12 Middle Occipital Gyrus R

-9 -84 30 Cuneus L

21 -81 30 Superior Occipital Gyrus R

9 -69 45 Precuneus R

6 -54 66 Precuneus R

-57 -15 -3 Middle Temporal Gyrus L

-48 -36 24 Supra Marginal Gyrus L

-36 -78 -12 Inferior Occipital Gyrus L

9 -90 9 Cuneus R

//Hu et al, 2014

//Subjects=52

6 -54 -33 Anterior Cerebellum Lobe R

36 51 -3 Middle Frontal Gyrus R

-18 -42 69 Postcentral Gyrus L

-12 -57 -3 Lingual Gyrus L

-21 -72 -9 Lingual Gyrus L

24 -6 0 Pallidum R

18 -45 66 Postcentral Gyrus R

-33 -75 45 Inferior Parietal Gyrus L

51 -24 15 Superior Temporal Gyrus R

-9 -84 24 Cuneus L

-15 30 -15 Inferior Frontal Gyrus L

3 -66 36 Precuneus R

-15 33 24 Anterior Cingulate Cortex L

-1 -84 18 Cuneus L

24 18 0 Putamen R

72 -36 -3 Middle Temporal Gyrus R

-3 -84 0 Lingual Gyrus L

-9 24 -12 Rectus Gyrus L

48 6 -18 Middle Temporal Gyrus R

9 -36 72 Paracentral Lobule R

-27 -51 57 Superior Parietal Gyrus L

-42 -30 60 Postcentral Gyrus L

57 -9 48 Postcentral Gyrus R

-12 -12 42 Middle Cingulate Cortex L

-33 -39 42 Inferior Parietal Gyrus L

63 0 21 Postcentral Gyrus R

//Kagerer et al, 2011

//Subjects=21

0 -24 -39 Brain Stem

0 -31 28 Posterior Cingulate Gyrus

-30 26 4 Insular Cortex L

-12 11 -11 Nucleus Accumbens L

12 14 -8 Nucleus Accumbens R

-3 8 -5 Hypothalamus

-12 -34 7 Thalamus L

18 -25 16 Thalamus R

-12 38 -5 Medial Orbitofrontal Cortex L

3 -94 16 Occipital Pole

-12 17 -8 Nucleus Accumbens L

-3 8 -5 Hypothalamus

18 -25 16 Thalamus R

9 -91 -5 Occipital Pole

-39 -1 7 Insular Cortex L

42 5 -5 Insular Cortex R

-24 -4 -14 Amygdala L

3 2 34 Anterior Cingulate Gyrus

//Manzouri and Savic, 2018

//Subjects=70

-6 -58 18 Precuneus L

4 -73 16 Cuneus R

11 35 12 Rostral Anterior Cingulate R

15 51 3 Anterior Cingulate Cortex

11 -48 6 Precuneus

//Paul et al, 2008

//Subjects=24

3 -3 9 Hypothalamus R

9 39 -18 Orbitofrontal R

34 -57 0 Parietal R

-34 57 0 Parietal L

-6 48 -18 Orbitofrontal L

//Ponseti et al, 2009

//Subjects=26

-62 -20 0 Insula L

20 50 22 Superior Frontal Gyrus R

34 -16 -8 Insula R

-16 -46 46 Posterior Cingular Gyrus L

44 -14 40 Dorsal Premotor Cortex R

10 -50 38 Posterior Cingulate Cortex R

26 -70 56 Superior Parietal Lobule R

4 -26 6 Posterior Thalamus M

//Safron et al, 2017

//Subjects=45

0 17 -8 Ventral Striatum

//Zhang et al, 2011

//Subjects=32

-1 39 -12 Medial Frontal Gyrus L

-1 -81 36 Cuneus L

Homosexual_vs_Heterosexual Opposite Sex

//Manzouri and Savic, 2018

//Subjects=70

-42 -81 2 Lateral, Occipital and Parietal Cortex L

//Manzouri and Savic, 2018

//Subjects=70

-60 -33 -8 Superior and Middle Temporal Cortex L

-14 40 16 Superior Frontal Cortex L

-8 -83 4 Pericalcarine Cortex L

-13 -57 18 Precuneus Cortex L

-34 33 8 Rostral Middle Frontal Cortex L

-48 -25 -7 Superior Temporal Cortex L

5 -57 26 Precuneus Cortex L

53 -28 -14 Inferior Temporal Cortex R

-15 56 17 Anterior Cingulate Cortex

//Safron et al, 2018

//Subjects=38

0 17 -8 Ventral Striatum

Homosexual_vs_Heterosexual Opposite & Assigned Sex at Birth

//Perry et al, 2017

//Subjects=52

-47 -30 23 Temporal Parietal Junction

**Appendix 5 – Risk of Bias_Gender Identity Research (From Quadas tool)**

|  | **Item 1** | **Item 2** | **Item 3** | **Item 4** | **Item 5** | **Item 6** | **Item 7** | **Item 8** | **Item 9** | **Item 10** | **Item 11** | **Item 12** | **Item 13** | **Item 14** |
| --- | --- | --- | --- | --- | --- | --- | --- | --- | --- | --- | --- | --- | --- | --- |
| **Berglund et al. 2008** | No | Yes | / | / | / | / | / | Yes | / | / | / | / | Yes | - |
| **Burke et al. 2014** | No | Yes | / | / | / | / | / | Yes | / | / | / | / | Yes | Yes |
| **Burke et al. 2016** | No | Yes | / | / | / | / | / | Yes | / | / | / | / | Yes | - |
| **Clemens et al. 2017** | No | No | / | / | / | / | / | Yes | / | / | / | / | Yes | Yes |
| **Feusner et al. 2017** | No | Yes | / | / | / | / | / | Yes | / | / | / | / | Yes | - |
| **Gizewski et al. 2009** | No | Yes | / | / | / | / | / | Yes | / | / | / | / | Yes | - |
| **Hahn et al. 2015** | No | No | / | / | / | / | / | Yes | / | / | / | / | Yes | - |
| **Hoekzema et al. 2015** | No | No | / | / | / | / | / | Yes | / | / | / | / | Yes | - |
| **Junger et al. 2014** | No | No | / | / | / | / | / | Yes | / | / | / | / | Yes | - |
| **Kranz et al. 2014a** | No | No | / | / | / | / | / | Yes | / | / | / | / | Yes | Yes |
| **Kranz et al. 2014b** | No | No | / | / | / | / | / | Yes | / | / | / | / | Yes | - |
| **Kranz et al. 2015** | No | No | / | / | / | / | / | Yes | / | / | / | / | Yes | - |
| **Kranz et al. 2018** | No | No | / | / | / | / | / | Yes | / | / | / | / | Yes | - |
| **Ku et al. 2013** | No | No | / | / | / | / | / | Yes | / | / | / | / | Yes | - |
| **Lin et al. 2014** | No | Yes | / | / | / | / | / | Yes | / | / | / | / | Yes | - |
| **Luders et al. 2009** | No | Yes | / | / | / | / | / | No | / | / | / | / | No | - |
| **Manzouri et al. 2017** | No | No | / | / | / | / | / | Yes | / | / | / | / | Yes | - |
| **Nawata et al. 2010** | No | Yes | / | / | / | / | / | Yes | / | / | / | / | Yes | - |
| **Nota et al. 2017** | No | Yes | / | / | / | / | / | Yes | / | / | / | / | Yes | - |
| **Pol et al. 2006** | No | Yes | / | / | / | / | / | Yes | / | / | / | / | Yes | Yes |
| **Rametti et al. 2011a** | No | Yes | / | / | / | / | / | Yes | / | / | / | / | Yes | - |
| **Rametti et al. 2011b** | No | Yes | / | / | / | / | / | No | / | / | / | / | No | - |
| **Santarnecchi et al. 2012** | No | Yes | / | / | / | / | / | Yes | / | / | / | / | Yes | - |
| **Savic and Arver 2011** | No | Yes | / | / | / | / | / | Yes | / | / | / | / | Yes | Yes |
| **Schöning et al. 2010** | No | No | / | / | / | / | / | Yes | / | / | / | / | Yes | - |
| **Simon et al. 2013** | No | No | / | / | / | / | / | Yes | / | / | / | / | Yes | - |
| **Soleman et al. 2013** | No | Yes | / | / | / | / | / | No | / | / | / | / | No | - |
| **Spies et al. 2016** | No | No | / | / | / | / | / | No | / | / | / | / | No | - |
| **Yokota et al. 2005** | No | No | / | / | / | / | / | Yes | / | / | / | / | Yes | - |
| **Zubiaurre-Elorza et al. 2013** | No | Yes | / | / | / | / | / | Yes | / | / | / | / | Yes | - |

**Appendix 6 – Risk of Bias_Sexual Orientation Research (From Quadas tool)**

|  | **Item 1** | **Item 2** | **Item 3** | **Item 4** | **Item 5** | **Item 6** | **Item 7** | **Item 8** | **Item 9** | **Item 10** | **Item 11** | **Item 12** | **Item 13** | **Item 14** |
| --- | --- | --- | --- | --- | --- | --- | --- | --- | --- | --- | --- | --- | --- | --- |
| **Abé et al. 2014** | No | Yes | / | / | / | / | / | Yes | / | / | / | / | Yes | - |
| **Berglund et al. 2006** | No | Yes | / | / | / | / | / | Yes | / | / | / | / | Yes | - |
| **Hu et al. 2013** | No | Yes | / | / | / | / | / | Yes | / | / | / | / | Yes | - |
| **Hu et al. 2014** | No | Yes | / | / | / | / | / | Yes | / | / | / | / | Yes | - |
| **Hu et al. 2011** | No | Yes | / | / | / | / | / | Yes | / | / | / | / | Yes | - |
| **Hu et al. 2008** | No | Yes | / | / | / | / | / | Yes | / | / | / | / | Yes | - |
| **Kagerer et al. 2011** | No | No | / | / | / | / | / | Yes | / | / | / | / | Yes | - |
| **Kinnunen et al. 2004** | No | No | / | / | / | / | / | No | / | / | / | / | No | - |
| **Manzouri and Savic 2018** | No | No | / | / | / | / | / | Yes | / | / | / | / | Yes | - |
| **Paul et al. 2008** | No | Yes | / | / | / | / | / | Yes | / | / | / | / | Yes | - |
| **Perry et al. 2013** | No | Yes | / | / | / | / | / | Yes | / | / | / | / | Yes | - |
| **Ponseti et al. 2009** | No | Yes | / | / | / | / | / | Yes | / | / | / | / | Yes | - |
| **Ponseti et al. 2007** | No | Yes | / | / | / | / | / | Yes | / | / | / | / | Yes | - |
| **Safron et al. 2017** | No | Yes | / | / | / | / | / | Yes | / | / | / | / | Yes | Yes |
| **Safron et al. 2018** | No | Yes | / | / | / | / | / | Yes | / | / | / | / | Yes | Yes |
| **Savic et al. 2005** | No | No | / | / | / | / | / | Yes | / | / | / | / | Yes | - |
| **Savic and Lindström 2008** | No | No | / | / | / | / | / | Yes | / | / | / | / | Yes | - |
| **Sylva et al. 2013** | No | No | / | / | / | / | / | Yes | / | / | / | / | Yes | - |
| **Witelson et al. 2008** | No | Yes | / | / | / | / | / | Yes | / | / | / | / | Yes | - |
| **Zeki and Romaya 2010** | No | No | / | / | / | / | / | Yes | / | / | / | / | Yes | Yes |
| **Zhang et al. 2011** | No | Yes | / | / | / | / | / | Yes | / | / | / | / | Yes | - |
